# ZDHHC14-Mediated TEAD4 Palmitoylation Drives Th17 Cell Recruitment in Renal Immunopathology

**DOI:** 10.34133/research.0954

**Published:** 2025-10-16

**Authors:** Yena Zhou, Chuyue Zhang, Jikai Xia, Shunlai Shang, Qun Liu, Xinru Guo, Jie Zhang, Shaoyuan Cui, Xu Wang, Ran Liu, Yingjie Zhang, Lingling Wu, Quan Hong, Xiaoniao Chen, Ying Zheng

**Affiliations:** ^1^ Senior Department of Nephrology, Chinese PLA General Hospital, State Key Laboratory of Kidney Diseases, National Clinical Research Center for Kidney Diseases, Key Disciplines of National Administration of Traditional Chinese Medicine (zyyzdxk-2023310), Beijing 100853, China.; ^2^ School of Medicine, Nankai University, Tianjin 300071, China.; ^3^ Department of Nephrology, Institute of Kidney Diseases, West China Hospital of Sichuan University, Chengdu 610041, China.; ^4^ Department of Nephrology, China-Japan Friendship Hospital, Beijing 100029, China.; ^5^ Senior Department of Ophthalmology, Chinese PLA General Hospital, Beijing 100039, China.

## Abstract

Palmitoylation, a crucial posttranslational protein modification, plays an undefined role in immune-mediated kidney diseases. This study reveals a novel mechanism whereby palmitoylation regulates the activity of transcription factor TEAD4 to facilitate T helper 17 (Th17) cell recruitment in IgA nephropathy (IgAN), suggesting that inhibition of palmitoylation could serve as a “brake” mechanism to impede disease progression. By analyzing samples from IgAN patients and mouse models, we identified a marked positive correlation between the extent of Th17 cell infiltration in renal tissues and disease severity. Mechanistically, under inflammatory conditions, injured tubular epithelial cells up-regulate CCL20 expression through the transcription factor TEAD4, thereby facilitating Th17 cell recruitment. Notably, TEAD4 activity is regulated by palmitoylation modification rather than changes in protein expression levels. Further analysis identified ZDHHC14 as the key palmitoyltransferase mediating TEAD4 palmitoylation, which is highly expressed in renal tissues of both IgAN patients and model mice. Knockdown of ZDHHC14 effectively reduced CCL20 expression and subsequent Th17 cell infiltration. In vivo therapeutic experiments demonstrated that administration of the ZDHHC inhibitor 2-BP effectively attenuated Th17 cell infiltration and renal interstitial fibrosis in model mice, markedly delaying disease progression. This study provides the first evidence of TEAD4 palmitoylation-mediated regulation in immune-mediated kidney and proposes a novel strategy to modulate Th17-driven disorders, with broad implications for autoimmune and fibrotic diseases.

## Introduction

IgA nephropathy (IgAN) represents one of the most frequently encountered glomerular pathologies globally. Notably, nearly a quarter to one-third of affected individuals advance to end-stage renal disease (ESRD) over the course of 2 to 3 decades following their initial diagnosis. Such disease progression contributes markedly to the worldwide healthcare burden [1–3]. Despite notable advancements in understanding the pathogenesis of IgAN in recent years, the molecular mechanisms underlying its progression to ESRD remain incompletely elucidated. Increasing evidence suggests that, beyond the classical mechanism of immune complex deposition, inflammatory injury in the tubulointerstitial compartment plays a critical role in disease progression [4–6].

Emerging evidence highlights the T helper 17 (Th17) cell/CCL20 chemokine axis as a pivotal pathway in this process, driving tubulointerstitial injury in various forms of chronic kidney disease (CKD), including IgAN [7,8]. Th17 cell-mediated immune injury is increasingly recognized as a key contributor to IgAN progression [9,10]. Studies have demonstrated a substantial increase in Th17 cells in both the peripheral blood and renal tissues of IgAN patients, with their effector cytokine, interleukin-17 (IL-17), positively correlating with the severity of CKD progression [11–13]. However, the precise mechanisms driving Th17 cell recruitment in IgAN remain unclear. The CCR6/CCL20 axis is known to be a critical pathway regulating Th17 cell migration, with CCR6 being uniquely paired with the chemokine CCL20 [14–17]. Nevertheless, the molecular mechanisms underlying the up-regulation of CCL20 expression require further investigation.

Posttranslational modifications (PTMs) of proteins play pivotal roles in immune regulation [18–20]. Among these, S-palmitoylation, a reversible lipid modification catalyzed by the ZDHHC family of palmitoyltransferases (PATs) and regulated by acyl-protein thioesterases (APTs), has garnered increasing attention [21–24]. To date, 24 members of the ZDHHC family have been identified, each exhibiting specific functions in various physiological and pathological processes [25]. Recent studies have shown that ZDHHC9 participates in kidney fibrosis by modulating the Wnt/β-catenin signaling pathway, suggesting that palmitoylation may play an important role in the progression of kidney diseases [26]. However, the role of palmitoylation in immune-mediated kidney injury remains unexplored. Specifically, emphasizing the unexplored role of protein palmitoylation in tubular immune dysregulation highlights a critical gap in our knowledge.

The TEAD family of transcription factors is known to play critical roles in diverse physiological and pathological processes, with its activity finely regulated by PTMs [27,28]. Notably, TEAD4 has been identified as being regulated by palmitoylation [29,30], yet its role and regulatory mechanisms in immune-mediated kidney diseases remain largely unknown. Specifically, whether TEAD4 is involved in the regulation of CCL20 expression and the associated molecular mechanisms remains an important scientific question.

This study aims to elucidate the molecular mechanisms underlying Th17 cell infiltration in tubulointerstitial injury during IgAN progression and to investigate the role of palmitoylation in this process. We hypothesize that inflammation-damaged renal tubular epithelial cells (TECs) promote the expression of the chemokine CCL20 through palmitoylation-regulated transcription factors, thereby recruiting Th17 cells and exacerbating renal damage in IgAN. By analyzing clinical samples and BSA nephritis mice, combined with in vitro validation experiments, this study seeks to uncover the involvement of palmitoylation and its related molecular pathways in the pathogenesis of IgAN, providing novel strategies for targeted therapy in IgAN.

## Results

### Th17 cell infiltration positively correlates with the severity of IgAN-CKD in patients and mouse models

**Fig. 1.**
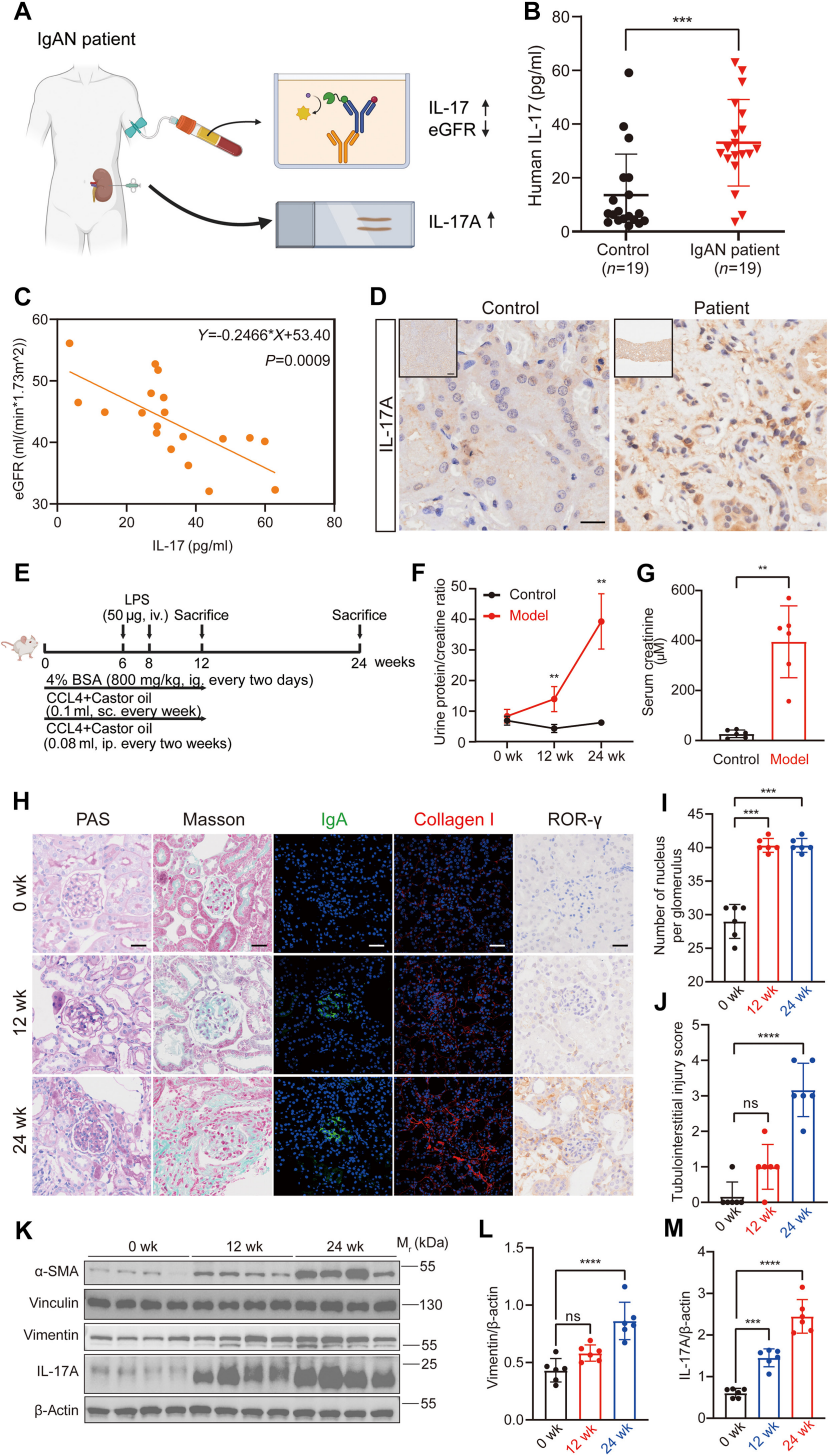
Th17 cell infiltration positively correlates with the severity of IgAN-CKD in patients and BSA nephritis mouse models. (A) Schematic representation of the sample collection workflow from IgAN-CKD patients, illustrating elevated IL-17 levels in both peripheral blood and renal tissue serum. (B) ELISA analysis of peripheral blood IL-17 levels in IgAN-CKD patients compared to healthy controls (*n* = 19). (C) Negative correlation between peripheral blood IL-17 levels and eGFR in IgAN-CKD patients (*n* = 19), indicating an association between IL-17 expression and CKD severity. (D) Representative immunohistochemical (IHC) images of renal tissues from IgAN-CKD patients and adjacent nontumorous tissues (*n* = 3), stained with anti-IL-17A antibody. Scale bars, 200 μm and 20 μm (enlargement). (E) The schematic outlines the experimental protocol employed to establish the BSA nephritis murine model. To mimic the progression toward chronic kidney disease and end-stage renal disease, mice were euthanized at both 12 and 24 weeks. (F and G) Urine protein-to-creatinine ratio and serum creatinine levels in BSA nephritis mice (*n* = 6). (H) Representative images of renal tissues from BSA nephritis mice stained with PAS, Masson, IF [anti-IgA antibody, green fluorescence, fluorescein isothiocyanate (FITC)-labeled; anti-collagen I antibody, red fluorescence, Cy3-labeled], and IHC (anti-ROR-γ antibody; *n* = 6; scale bar, 20 μm). (I) Mesangial cell numbers were quantitatively assessed in PAS sections, with analyses conducted across 6 biological replicates to ensure statistical robustness (*n* = 6). (J) Tubulointerstitial injury was evaluated using a semiquantitative scale ranging from 0 to 4, where a score of 0 denotes the absence of injury, 1 reflects involvement of less than 25% of the area, 2 corresponds to 25% to 50%, 3 corresponds to 50% to 75%, and 4 represents greater than 75% of the area affected (*n* = 6). (K) WB analysis of α-SMA, vimentin, and IL-17A protein expression, with β-actin and vinculin as loading controls (*n* = 6). (L and M) Semiquantitative analysis of WB results. Comparisons between 2 groups are performed using Student’s *t* test, whereas differences among 3 or more groups are assessed by one-way analysis of variance (ANOVA). All results are expressed as mean ± SD. Statistical significance is denoted as follows: ns, not significant; **P < 0.01; ***P < 0.001; ****P < 0.0001. The schematic diagrams in (A) and (E) are created using BioRender.com under a valid license.

To investigate the role of IL-17 in the progression of IgAN-CKD, we examined IL-17 expression in both human peripheral blood and kidney tissues (Fig. 1A). Serum samples were collected from IgAN patients with CKD stage 3 and age- and gender-matched healthy volunteers. Enzyme-linked immunosorbent assay (ELISA) analysis revealed a marked up-regulation of serum IL-17 levels in CKD stage 3 IgAN patients, which negatively correlated with eGFR (Fig. 1B and C). Furthermore, elevated IL-17 levels were observed in IgAN patients with tubulointerstitial lesions (Fig. 1D). In addition, analysis of transcriptomic data from the kidney tissues of IgAN patients revealed that the Th17 cell differentiation pathway was up-regulated, accompanied by the concurrent activation of fibrosis-related pathways (Fig. S1).

To further explore the association between IL-17 and disease progression, we established the BSA nephritis mouse model and collected kidney tissues at 12 and 24 weeks (Fig. 1E). As the disease progressed, the mice exhibited notable increases in blood urea nitrogen, serum creatinine, 24-h urinary protein excretion, and urinary protein-to-creatinine ratio (Fig. 1F and G). Histological analyses, including periodic acid–Schiff (PAS) and Masson staining, as well as immunofluorescence (IF) of IgA and immunohistochemistry (IHC) of ROR-γ, which is the key transcription factor of Th17 cell (Fig. 1H to J), demonstrated mesangial cell proliferation, mesangial matrix expansion, and IgA deposition in the glomeruli at 12 weeks, consistent with IgAN pathology. However, no marked tubulointerstitial damage or fibrosis was observed at this stage. By 24 weeks, glomerular lesions had worsened, and prominent tubulointerstitial fibrosis was evident, indicating progression to late-stage CKD. Notably, Th17 cell infiltration in the kidney increased substantially from 12 to 24 weeks, predominantly localized in the tubulointerstitial region, accompanied by elevated IL-17 expression. The Western blotting (WB) analysis is consistent with pathological findings (Fig. 1K to M). These findings suggest a positive correlation between Th17 cell/IL-17 levels and the progression of IgAN-CKD.

**Fig. 2.**
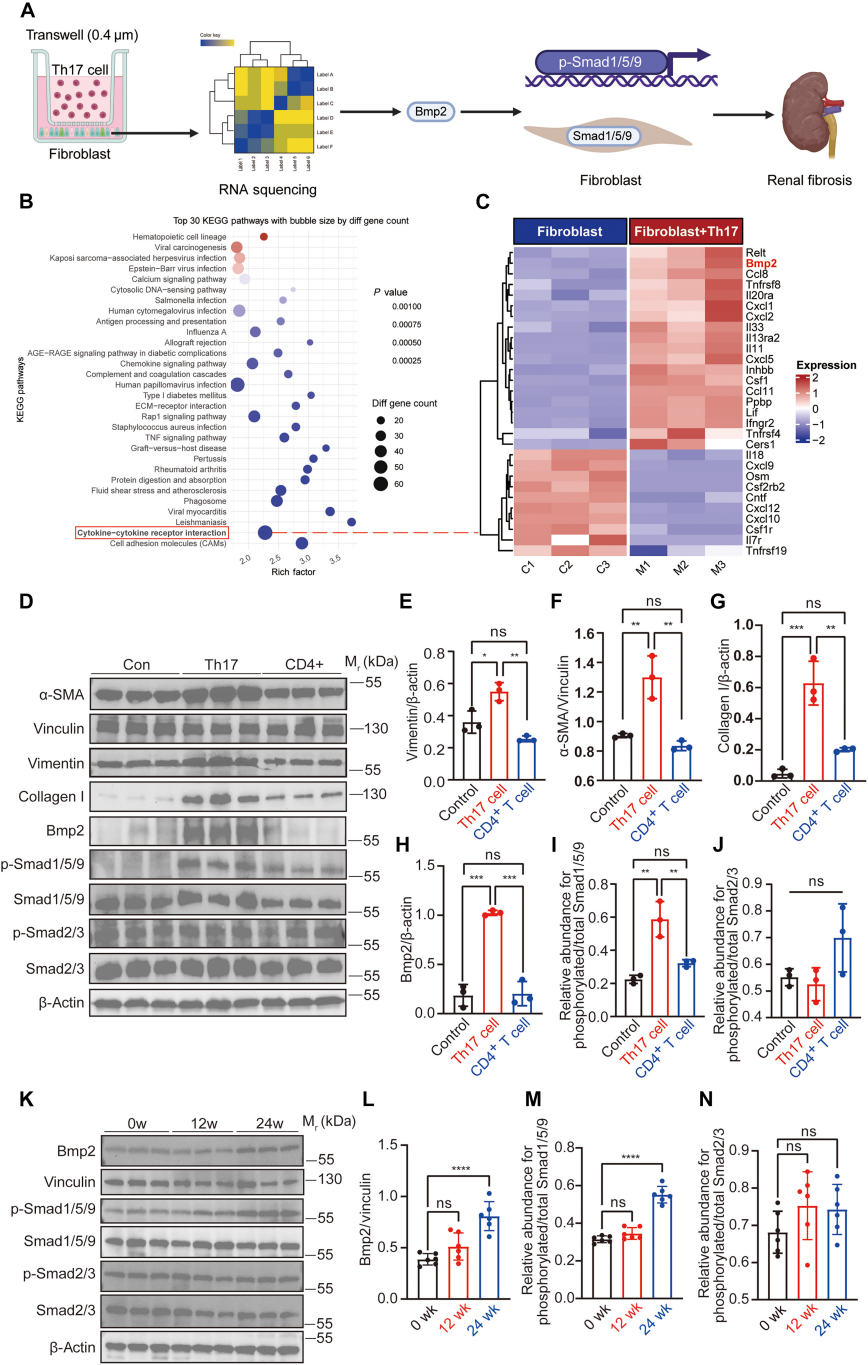
Th17 cell infiltration promotes renal fibrotic progression in BSA nephritis mice. (A) Schematic representation of the study design aimed at elucidating the profibrotic role of Th17 cells in renal fibrosis. (B and C) RNA-sequencing analysis of fibroblasts cultured alone or cocultured with Th17 cells reveals a marked up-regulation of Bmp2 expression in fibroblasts cocultured with Th17 cells (*n* = 3). (D to J) WB analysis of fibroblasts cultured alone or cocultured with Th17 cells shows the expression levels of α-SMA, vimentin, type I collagen, Bmp2, p-Smad1/5/9, and p-Smad2/3 proteins. β-Actin and vinculin serve as internal controls. The corresponding semiquantitative data are depicted on the right (*n* = 3). (K to N) WB analysis shows the protein expression levels of Bmp2, p-Smad1/5/9, and p-Smad2/3 in renal tissues from BSA nephritis mice, with β-actin and vinculin serving as loading controls. Semiquantitative results are shown on the right (*n* = 6). Comparisons between 2 groups are performed using Student’s *t* test, whereas differences among 3 or more groups are assessed by one-way ANOVA. All results are expressed as mean ± SD. Statistical significance is denoted as follows: ns, not significant; *P < 0.05; **P < 0.01; ***P < 0.001; ****P < 0.0001. The schematic diagram in (A) is created using BioRender.com under a valid license.

To elucidate the profibrotic effects of Th17 cells, we conducted in vitro coculture experiments with Th17 cells and fibroblasts (Fig. 2A). Coculture with Th17 cells led to a marked increase in the expression of fibrosis-related markers, including Bmp2, in fibroblasts (Fig. 2B and C). Fibroblasts cocultured with Th17 cells exhibited a fibrotic phenotype, characterized by increased Bmp2 expression and Smad1/5/9 phosphorylation (Fig. 2D to J). Consistent with these findings, WB analysis of kidney tissues from the bovine serum albumin (BSA) nephritis mice, an animal model of IgAN, revealed a progressive increase in Bmp2 expression and phosphorylated Smad1/5/9 levels as CKD advanced (Fig. 2K to N). These results indicate that Th17 cell infiltration in the tubulointerstitial region exacerbates IgAN-CKD progression by activating fibroblasts through the Bmp2/Smad1/5/9 signaling pathway. In the BSA nephritis mice, the progression from 12 to 24 weeks was accompanied by increased Th17 cell infiltration, up-regulated IL-17 expression, and marked tubulointerstitial fibrosis. The in vitro findings further confirmed that Th17 cells promote fibroblast activation via the Bmp2/Smad1/5/9 pathway, thereby accelerating CKD progression.

### Chemokine CCL20 recruits Th17 cells and is regulated by the transcription factor TEAD4

**Fig. 3.**
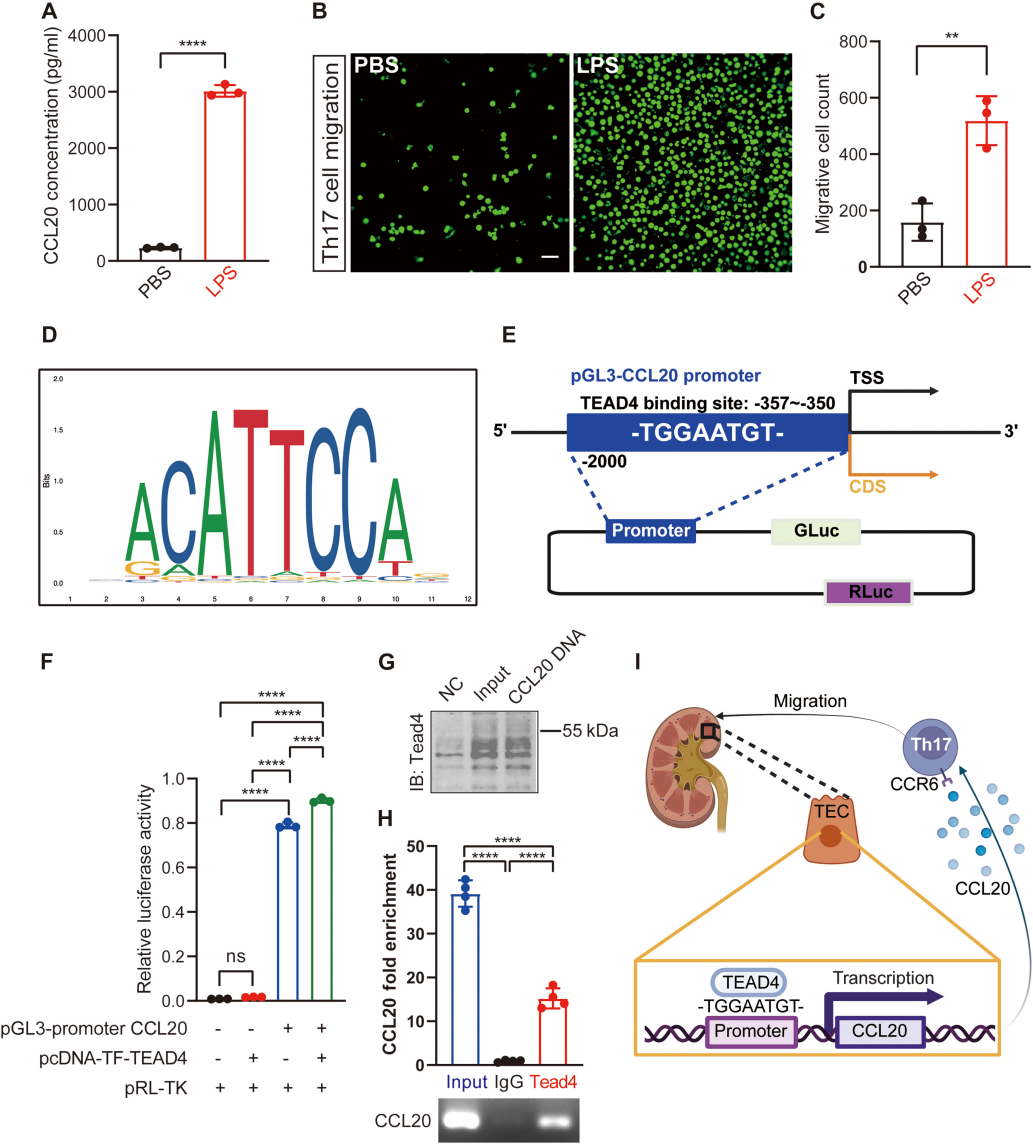
Chemokine CCL20 recruits Th17 cells and is regulated by the transcription factor TEAD4. (A) ELISA results demonstrate elevated CCL20 expression levels in inflamed renal tubular epithelial cells (TECs) compared to normal TECs (*n* = 3). (B and C) Transwell migration assays revealed a marked increase in the number of Th17 cells migrating toward inflamed renal TECs, as visualized by calcein staining (green fluorescence; *n* = 3; scale bar, 50 μm). The graph on the right quantifies the average number of migrating Th17 cells per field. (D) JASPAR analysis predicted potential TEAD4 binding sites within the promoter region of CCL20, suggesting a regulatory interaction. (E) Schematic representation of the construction of TEAD4 overexpression vectors and CCL20 promoter luciferase reporter gene vectors. (F) Luciferase reporter assays in 293T cells showed that TEAD4 overexpression substantially enhances CCL20 promoter activity, supporting its role as a transcriptional activator (*n* = 3). (G) DNA pull-down assays subsequently confirmed a direct physical interaction between the CCL20 promoter and TEAD4 protein. (H) This binding was further validated by chromatin immunoprecipitation (ChIP) assays, which demonstrated the specific recruitment of TEAD4 to the endogenous CCL20 promoter region (*n* = 3). (I) Proposed mechanism illustrating how TEAD4 promotes Th17 cell recruitment by up-regulating CCL20 transcription. Comparisons between 2 groups are performed using Student’s *t* test, whereas differences among 3 or more groups are assessed by one-way ANOVA. All results are expressed as mean ± SD. Statistical significance is denoted as follows: ns, not significant; **P < 0.01; ****P < 0.0001. The schematic diagrams in (E) and (I) are created using BioRender.com under a valid license.

To investigate the mechanisms underlying the increased Th17 cell infiltration, we focused on the chemokine CCL20, which is known to recruit Th17 cells [31]. However, the specific mechanism driving CCL20 up-regulation remained unclear. Given that Th17 cells predominantly infiltrate the tubulointerstitial region, we hypothesized that inflamed renal TECs might produce elevated levels of CCL20. To test this, we established an in vitro inflammation model by stimulating primary mouse TECs (pmTECs) with varying concentrations of lipopolysaccharide (LPS), which recapitulated a pan-inflammatory environment. A concentration of 50 μg/ml was selected as optimal for inducing inflammation (Fig. S2). LPS-stimulated pmTECs exhibited substantially increased CCL20 expression (Fig. 3A). When cocultured with Th17 cells, LPS-damaged pmTECs enhanced Th17 cell migration compared to normal pmTECs (Fig. 3B and C). These findings suggest that inflamed TECs produce elevated levels of CCL20, thereby recruiting more Th17 cells.

To further elucidate the mechanism of CCL20 up-regulation, we performed transcription factor prediction using the UCSC and JASPAR databases. TEAD4 emerged as a top candidate transcription factor, predicted to bind the CCL20 promoter region with high affinity (Fig. 3D and Table S4; binding score: 14.6). Dual-luciferase reporter assays confirmed that TEAD4 overexpression directly binds to the CCL20 promoter and enhances its transcription (Fig. 3E and F). Furthermore, this direct physical interaction was definitively confirmed through both DNA pull-down and chromatin immunoprecipitation (ChIP) assays (Fig. 3G and H). These results indicate that inflammation-induced TEAD4 binds to the CCL20 promoter, leading to increased CCL20 expression (Fig. 3I). Collectively, our findings demonstrate that inflamed renal TECs up-regulate CCL20 via TEAD4, thereby promoting Th17 cell recruitment and exacerbating IgAN-CKD progression.

### Renal tubular inflammation instigates a TEAD4 regulatory switch, shifting CCL20 control from abundance to palmitoylation-dependent activity

**Fig. 4.**
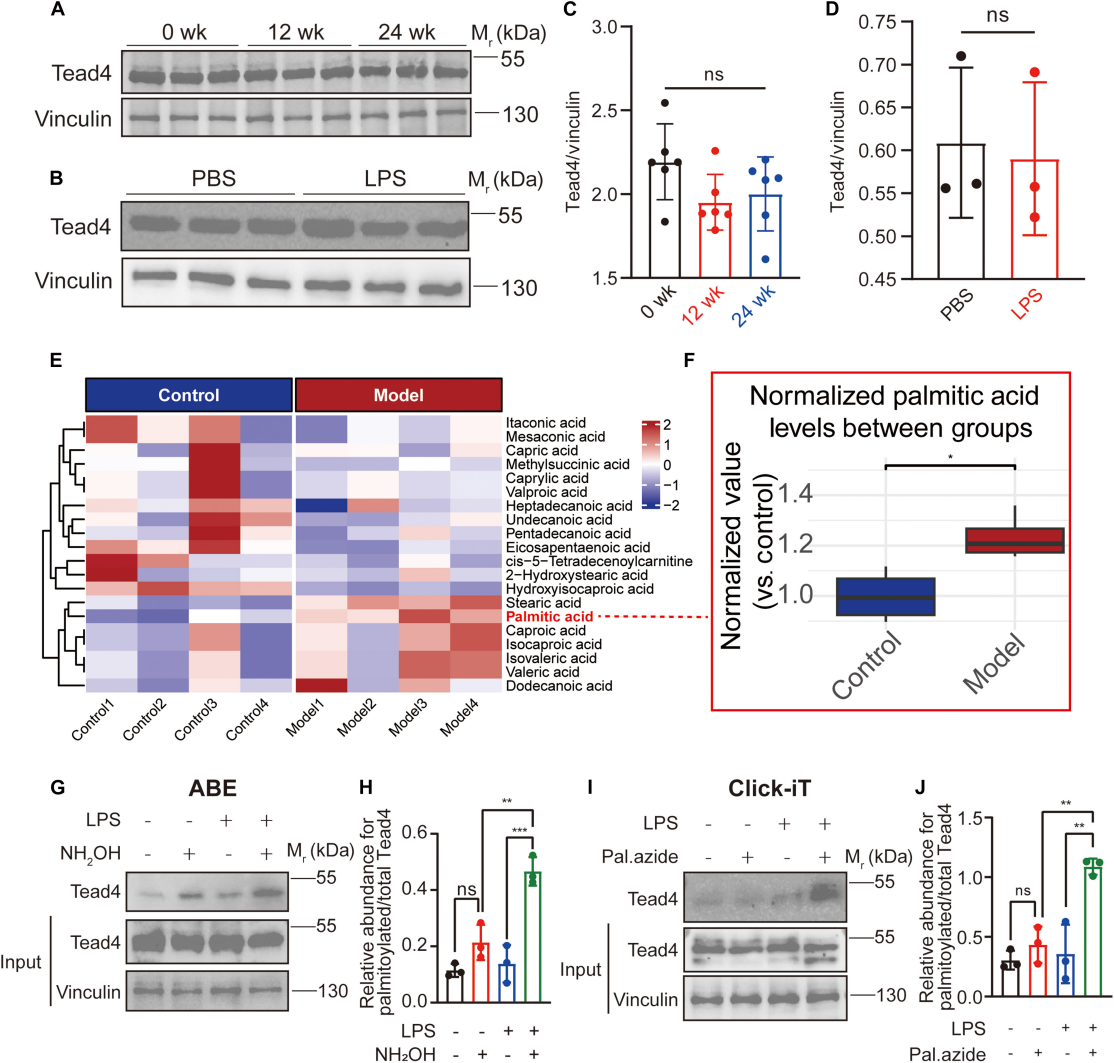
Palmitoylation, rather than protein expression, predominantly regulates TEAD4 activity. (A and B) WB analysis revealed no marked difference in TEAD4 protein expression levels between BSA nephritis mice and normal mouse kidneys (*n* = 6) or between inflamed and normal renal TECs (*n* = 3). Vinculin was used as a loading control. (C and D) Semiquantitative analysis of WB results from (A) and (B), confirming the lack of marked changes in TEAD4 protein expression. (E and F) Metabolomic analysis of normal and BSA nephritis mouse kidneys revealed marked differences in palmitic acid content (*n* = 4). (G to J) ABE and Click-iT assays demonstrated a substantial increase in TEAD4 palmitoylation levels in inflamed renal TECs. Semiquantitative analysis of palmitoylation levels is shown on the right (*n* = 3). Comparisons between 2 groups are performed using Student’s *t* test, whereas differences among 3 or more groups are assessed by one-way ANOVA. All results are expressed as mean ± SD. Statistical significance is denoted as follows: ns, not significant; *P < 0.05; **P < 0.01; ***P < 0.001.

To elucidate the mechanism by which TEAD4 regulates CCL20 transcription, we first examined TEAD4 expression levels in kidney tissues and pmTECs under inflammatory conditions. Interestingly, no obvious increase in TEAD4 expression was observed in the injury group (Fig. 4A to D), suggesting that the TEAD4’s control over CCL20 transcription dynamically switches from being abundance-dependent at baseline to being activity-driven via PTM during inflammation. Previous investigations have elucidated that palmitoylation serves as a pivotal PTM essential for both the stabilization and activation of TEAD4. Specifically, TEAD4 mutants deficient in palmitoylation exhibit pronounced instability and are subject to accelerated proteasomal degradation, a process orchestrated by the E3 ubiquitin ligase ChIP [29,30,32]. To determine if the necessary conditions for TEAD4 palmitoylation existed in vivo, we performed metabolomic analysis on renal tissues from our BSA nephritis model. This revealed a marked elevation of palmitic acid in the inflamed kidneys (Fig. 4E and F). Palmitate, as a predominant saturated fatty acid accounting for approximately 20% to 30% of the total fatty acid composition in the human body, can originate either from dietary intake or through endogenous synthesis via de novo lipogenesis (DNL) [33,34]. Given its role as a substrate for protein palmitoylation, the elevation of palmitate levels implies enhanced palmitoylation activity within inflamed kidney tissues, which may consequently augment the biological functions of particular proteins [35]. Consistent with this, Click-iT assays on primary TECs isolated directly from the model mice confirmed that TEAD4 undergoes palmitoylation in vivo (Fig. S3A and B). We then conducted acyl-biotin exchange (ABE) and Click-iT assays, both of which demonstrated that TEAD4 is indeed palmitoylated, with increased palmitoylation levels observed in pmTECs following inflammatory injury (Fig. 4G to J).

**Fig. 5.**
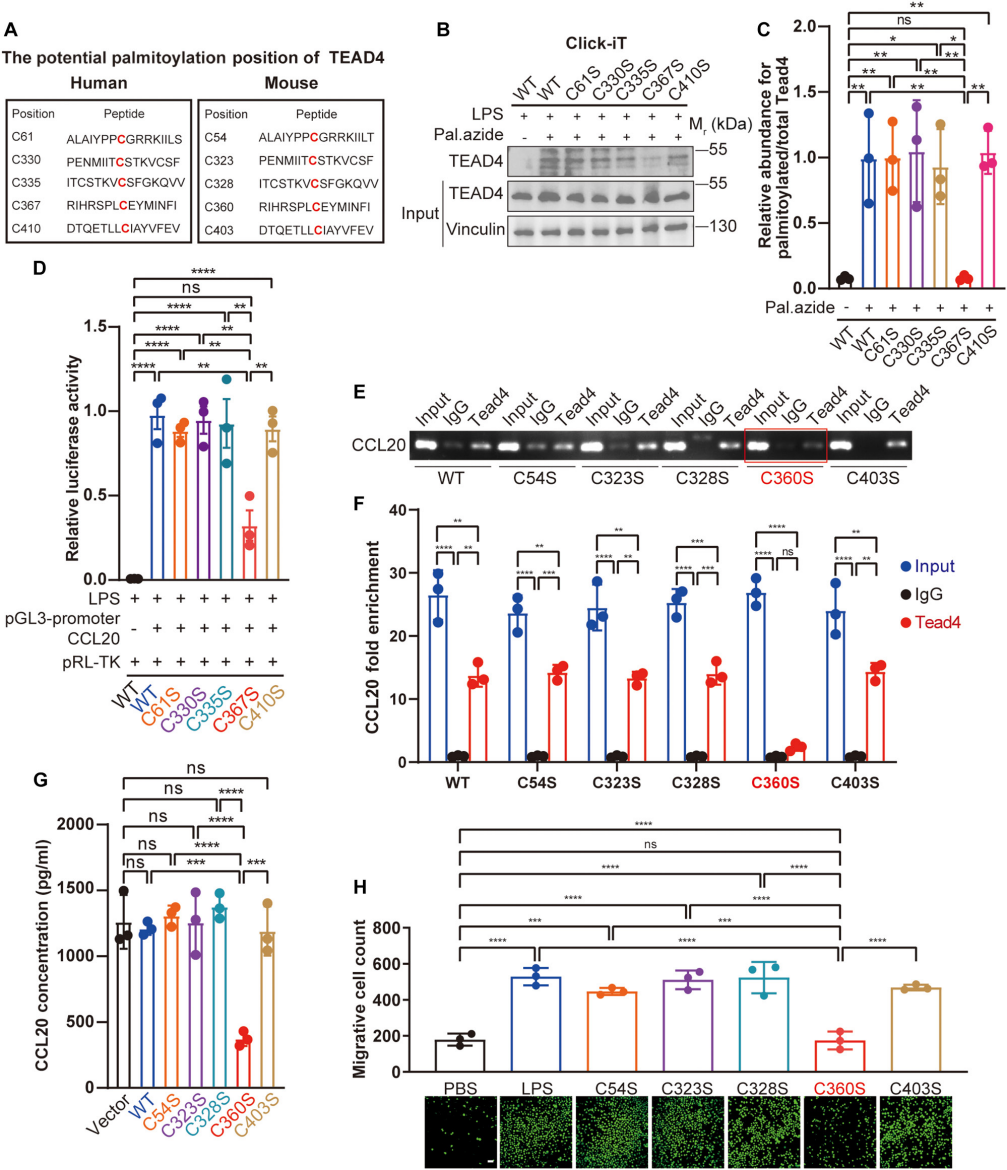
Palmitoylation of TEAD4 at cysteine 367 (C367) is critical for its transcriptional regulation of CCL20. (A) Probable palmitoylation sites of TEAD4 in humans and mice. (B and C) The Click-iT assay revealed that the C367S (human) mutation in TEAD4 completely abrogated its palmitoylation (*n* = 3). (D) The dual-luciferase reporter assays revealed that the C367S (human) mutation in TEAD4 reduced its transcriptional regulation of CCL20 (*n* = 3). (E and F) ChIP followed by RT-qPCR analysis revealed that the C360S (mouse) mutation substantially diminished the binding occupancy of TEAD4 at the endogenous CCL20 promoter region (*n* = 3). (G) Consistent with this transcriptional defect, ELISA confirmed that the C360S (mouse) mutation results in substantially decreased CCL20 protein expression (*n* = 3). (H) As a direct functional consequence, Transwell migration assays demonstrated that expression of the TEAD4-C360S mutant in renal TECs markedly impaired the recruitment of Th17 cells (calcein AM staining, green fluorescence; *n* = 3; scale bar, 50 μm). Differences among 3 or more groups are assessed by one-way ANOVA. All results are expressed as mean ± SD. Statistical significance is denoted as follows: ns, not significant; *P < 0.05; **P < 0.01; ***P < 0.001; ****P < 0.0001.

To identify potential palmitoylation sites on TEAD4, we utilized the CSS-Palm 4.0 software for predictive analysis. This computational approach revealed that cysteine residues 61, 330, 335, 367, and 410 of TEAD4 are likely candidates for palmitoylation modifications. Notably, these residues are highly conserved between humans and mice, underscoring their potential functional significance (Fig. 5A). To validate these predictions, we conducted Click-iT assays, which demonstrated that a single C367S mutation almost entirely abrogated TEAD4 palmitoylation (Fig. 5B and C). Furthermore, this mutation substantially diminished TEAD4’s ability to regulate the transcription of CCL20 (Fig. 5D). This finding was further supported by ChIP and reverse transcription quantitative polymerase chain reaction (RT-qPCR) assays (Fig. 5E and F). Functionally, this single-residue substitution resulted in a cascade of downstream effects: It markedly reduced CCL20 expression (Fig. 5G) and attenuated the chemotactic migration of Th17 cells (Fig. 5H). Collectively, these results strongly indicate that C367 (human)/C360 (mouse) is the principal and functionally critical palmitoylation site of TEAD4.

### Fatty acid synthase regulates palmitic acid synthesis, providing substrate for TEAD4 palmitoylation

**Fig. 6.**
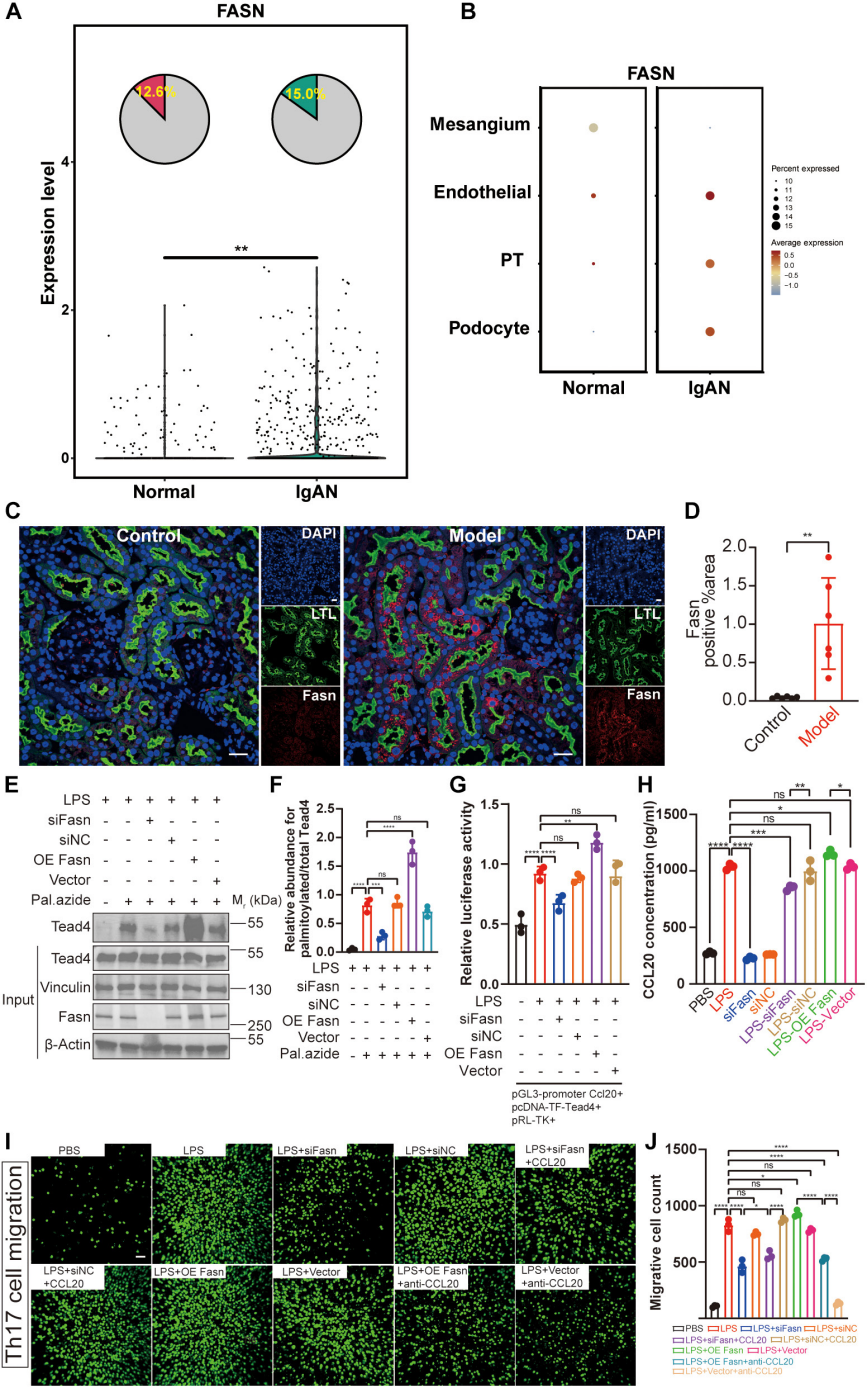
Fatty acid synthase (FASN) regulates palmitic acid synthesis, providing substrate for TEAD4 palmitoylation. (A and B) Single-cell sequencing analysis demonstrated differential FASN expression levels in renal tissues, including proximal tubular cells (PT cells), from IgAN patients. (C and D) Immunofluorescence (IF) staining showed the expression of FASN in kidneys of BSA nephritis mice (FASN, Cy3-labeled, red fluorescence; LTL, FITC-labeled, green fluorescence; *n* = 6; scale bar, 20 μm). Semiquantitative analysis of FASN-positive areas is presented on the right. (E and F) Click-iT assays revealed that TEAD4 palmitoylation was diminished in inflamed renal TECs following FASN knockdown, whereas, conversely, it was enhanced by FASN overexpression (*n* = 3). (G) Reflecting this change in modification, luciferase reporter assays showed that FASN knockdown impaired TEAD4-mediated transcriptional activation of the CCL20 promoter, while FASN overexpression potentiated it (*n* = 3). (H) Consistent with the transcriptional data, ELISA confirmed that CCL20 protein secretion was decreased following FASN knockdown and increased upon FASN overexpression (*n* = 3). (I and J) Transwell migration assays indicated that FASN knockdown markedly reduced the number of Th17 cells migrating toward inflamed renal TECs. Transwell migration assays indicated that FASN knockdown substantially reduced the number of Th17 cells migrating toward inflamed renal TECs; notably, this reduction was rescued by the addition of recombinant CCL20. Conversely, FASN overexpression augmented Th17 migration, an effect that was reversed by a CCL20 neutralizing antibody (calcein AM staining, green fluorescence; *n* = 3; scale bar, 50 μm). The graph on the right quantifies the average number of migrating cells per field. Comparisons between 2 groups are performed using Student’s *t* test, whereas differences among 3 or more groups are assessed by one-way ANOVA. All results are expressed as mean ± SD. Statistical significance is denoted as follows: ns, not significant; *P < 0.05; **P < 0.01; ***P < 0.001; ****P < 0.0001.

The palmitoylation of TEAD4 is modulated by the availability of palmitate, a saturated fatty acid that is synthesized de novo through the catalytic activity of fatty acid synthase (FASN) [29]. Notably, FASN serves as a central enzyme in fatty acid metabolism, facilitating the production of key saturated fatty acids, including both palmitate and stearate [36–39]. To determine whether TEAD4 in inflamed IgAN kidney tissues is subject to palmitoylation, we analyzed single-cell sequencing data from IgAN patient kidney tissues [40]. The results revealed high expression of the FASN gene, particularly in injured proximal TECs (Fig. 6A and B). This was further validated in BSA nephritis mice, where FASN was highly expressed and localized to TECs (Fig. 6C and D and Fig. S4A and B). Similarly, FASN expression was elevated in pmTECs under inflammatory conditions (Fig. S4C and D).

To directly interrogate the functional role of FASN in this pathway, we modulated its expression in our in vitro inflammation model. As hypothesized, genetic knockdown of FASN markedly diminished the level of TEAD4 palmitoylation, whereas its overexpression substantially enhanced this modification (Fig. 6E and F). Correspondingly, FASN knockdown impaired the ability of TEAD4 to drive CCL20 transcription, leading to reduced CCL20 expression. Conversely, FASN overexpression augmented this regulatory activity, resulting in elevated CCL20 levels (Fig. 6G and H). Ultimately, these molecular changes translated directly to a functional cellular phenotype. FASN depletion attenuated the chemotactic recruitment of Th17 cells by inflamed TECs, an effect that was completely rescued by the addition of exogenous CCL20 (100 ng/ml). In contrast, FASN overexpression potentiated Th17 migration, and this enhancement was markedly blunted by the introduction of a CCL20 neutralizing antibody (10 μg/ml) (Fig. 6I and J). Taken together, these results establish a complete pathological axis wherein inflammation-induced FASN synthesizes the palmitate required for TEAD4 palmitoylation, which in turn enhances its transcriptional activity to drive CCL20 expression and subsequent Th17 cell recruitment.

### ZDHHC14 is the key PAT promoting TEAD4 palmitoylation in IgAN

**Fig. 7.**
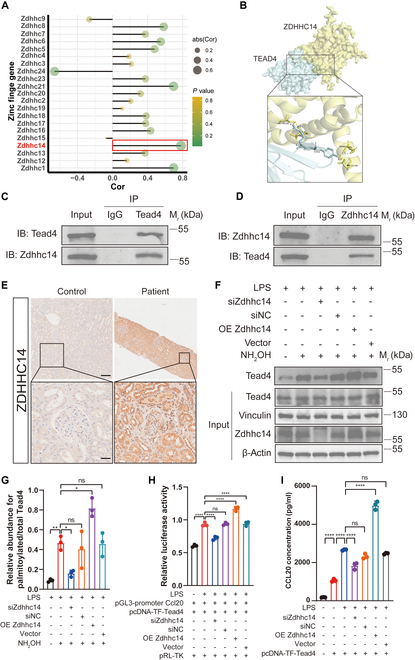
ZDHHC14 is the key palmitoyltransferase (PAT) promoting TEAD4 palmitoylation and is highly expressed in the kidneys of IgAN patients and BSA nephritis mice. (A) RNA-sequencing analysis revealed a marked up-regulation of ZDHHC14 expression in the renal tissues of BSA nephritis mice. (B) Molecular docking predicted potential binding sites and binding affinity between ZDHHC14 and TEAD4, suggesting a direct interaction. (C and D) Co-immunoprecipitation (Co-IP) assays confirmed the interaction between TEAD4 and ZDHHC14 in renal TECs following inflammatory injury. (E) IHC staining showed substantially higher ZDHHC14 expression in the renal tissues of IgAN patients compared to adjacent nontumor tissue (*n* = 3). Scale bars, 200 μm (upper panel) and 20 μm (lower panel). (F and G) ABE assays demonstrated that ZDHHC14 knockdown [via small interfering RNA (siRNA)] reduced, while ZDHHC14 overexpression increased, TEAD4 palmitoylation levels in inflamed renal TECs (*n* = 3). (H) Dual-luciferase reporter assays revealed that ZDHHC14 knockdown decreased, while ZDHHC14 overexpression enhanced, TEAD4-mediated transcriptional activity on the CCL20 promoter in inflamed renal TECs (*n* = 3). (I) ELISA results indicated that ZDHHC14 knockdown reduced, while ZDHHC14 overexpression increased, CCL20 levels in inflamed renal TECs (*n* = 3). Differences among 3 or more groups are assessed by one-way ANOVA. All results are expressed as mean ± SD. Statistical significance is denoted as follows: ns, not significant; *P < 0.05; **P < 0.01; ****P < 0.0001.

To further investigate the molecular mechanism underlying TEAD4 palmitoylation, we sought to identify the upstream PAT responsible for this modification. PATs, encoded by the Zdhhcs gene family, catalyze the attachment of palmitate to specific cysteine residues on target proteins [25]. To identify the specific enzyme, we first screened the expression of all Zdhhc family members in BSA nephritis mice and inflamed pmTECs. This revealed that Zdhhc14 was the only member whose expression was effectively up-regulated in both the kidneys of BSA nephritis mice and in inflamed pmTECs (Fig. S5A and B). This increase in ZDHHC14 protein was subsequently confirmed by WB in both in vivo and in vitro settings (Fig. S5C to F). Complementing these expression data, transcriptomic analysis revealed that among the 24 members of the ZDHHC family, ZDHHC14 exhibited the highest correlation with TEAD4 in the kidneys of BSA nephritis mice (Fig. 7A). Subsequent molecular docking and co-immunoprecipitation (Co-IP) experiments confirmed a direct physical interaction between TEAD4 and ZDHHC14 (Fig. 7B and D). Crucially, the clinical relevance of this axis was underscored by the finding that ZDHHC14 was also highly expressed in the kidneys of IgAN patients (Fig. 7E).

To functionally validate the role of ZDHHC14 in TEAD4 palmitoylation, we manipulated ZDHHC14 expression in pmTECs. As expected, knockdown of ZDHHC14 substantially reduced TEAD4 palmitoylation levels, while overexpression of ZDHHC14 enhanced this modification (Fig. 7F and G and Fig. S6A and B). To confirm the specificity of this enzymatic relationship, we also investigated ZDHHC1 and ZDHHC21, which showed some correlation with TEAD4. However, altering their expression levels had no discernible effect on TEAD4 palmitoylation, confirming that ZDHHC14 is the principal enzyme responsible (Fig. S7A to H). Correspondingly, ZDHHC14 knockdown attenuated TEAD4’s transcriptional activation of CCL20, leading to decreased CCL20 expression, whereas ZDHHC14 overexpression amplified TEAD4’s transcriptional activity, resulting in increased CCL20 expression (Fig. 7H and I). These results conclusively demonstrate that ZDHHC14 is the critical PAT responsible for TEAD4 palmitoylation, thereby modulating its transcriptional activity and contributing to the recruitment of Th17 cells.

### FASN and ZDHHC14 converge to obligatorily regulate TEAD4 palmitoylation, driving Th17 cell recruitment

Our preceding findings established that FASN, by controlling palmitate substrate availability, and ZDHHC14, as the catalyzing transferase, both govern TEAD4 palmitoylation. This prompted us to investigate whether these 2 components function cooperatively and are mutually indispensable for this process. To address this, we performed reciprocal overexpression and knockdown experiments in inflamed pmTECs. Strikingly, neither the overexpression of ZDHHC14 in FASN-deficient cells nor the overexpression of FASN in ZDHHC14-deficient cells could rescue or enhance the level of TEAD4 palmitoylation (Fig. S8A to H). These data, from both ABE and Click-iT assays, provide unequivocal evidence that FASN and ZDHHC14 are nonredundant and obligatory components of the TEAD4 palmitoylation machinery; the presence of both substrate and enzyme is absolutely required.

Given that FASN and ZDHHC14 have potentially broad cellular functions, we next sought to confirm that their ultimate effect on Th17 cell infiltration is channeled specifically through TEAD4. To this end, we knocked down TEAD4 in inflamed pmTECs and then attempted to rescue the resulting phenotype by overexpressing either FASN or ZDHHC14. As expected, TEAD4 depletion dramatically reduced CCL20 expression and the subsequent migration of Th17 cells toward the inflamed TECs (Fig. S9A, D, and E). Critically, overexpression of either FASN or ZDHHC14 was entirely incapable of restoring CCL20 expression or rescuing the Th17 migratory defect in these TEAD4-deficient cells (Fig. S9B, C, and F to H). These results provide definitive evidence that FASN and ZDHHC14 operate in a linear, obligatory pathway that converges on TEAD4 to drive CCL20-mediated Th17 cell infiltration, confirming TEAD4 as the critical and indispensable downstream effector of this proinflammatory signaling axis.

### The PAT inhibitor 2-BP reduces Th17 cell infiltration and delays CKD progression

**Fig. 8.**
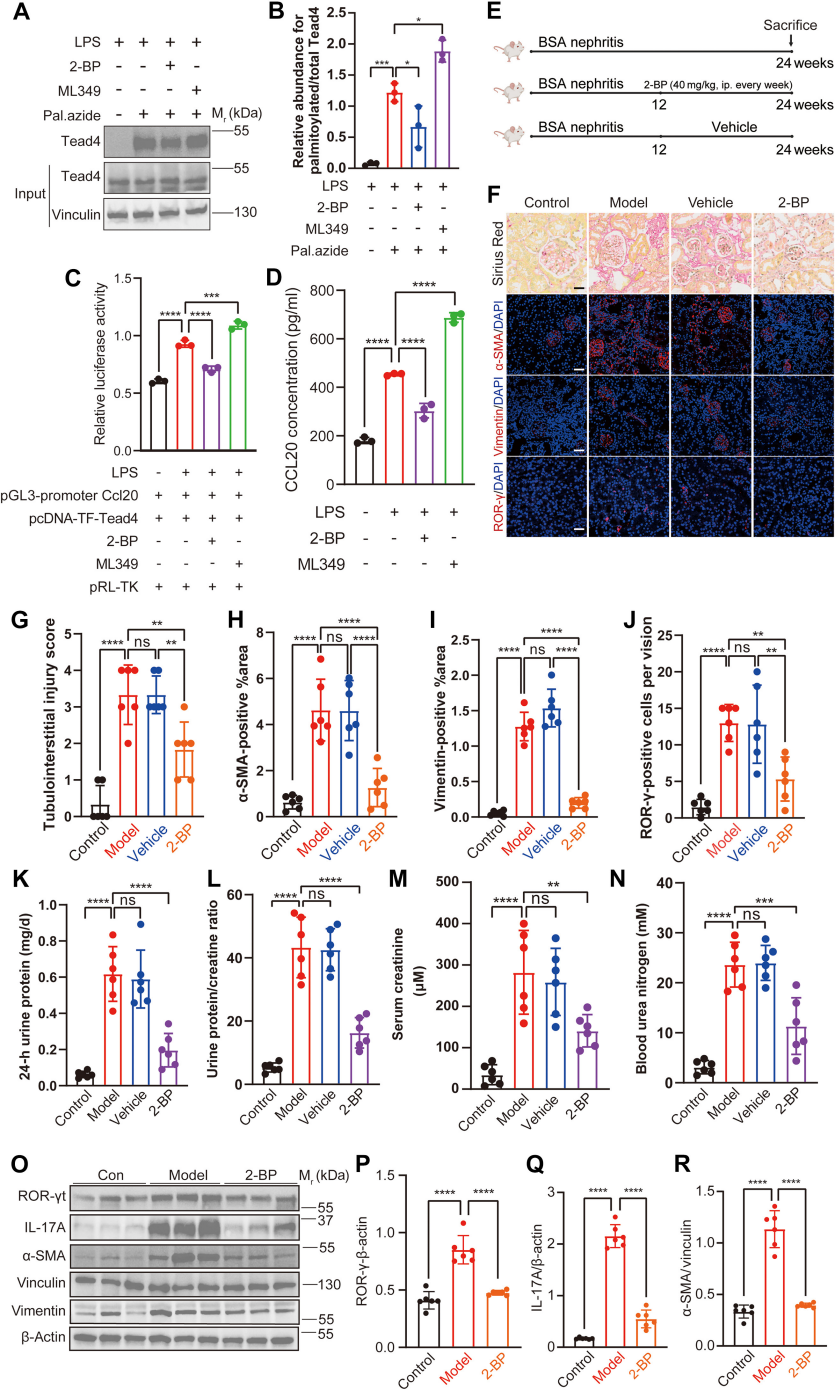
The PAT inhibitor 2-BP reduces Th17 cell infiltration and fibrosis progression in BSA nephritis mice. (A and B) To confirm their direct effects on TEAD4 modification, Click-iT assays showed that 2-BP treatment decreased TEAD4 palmitoylation, whereas, conversely, the depalmitoylase inhibitor ML349 enhanced its levels (*n* = 3). (C) Dual-luciferase reporter assays demonstrated that treatment with the PAT inhibitor 2-BP markedly decreased TEAD4-mediated transcriptional activity on the CCL20 promoter in inflamed renal TECs, whereas treatment with the depalmitoylase inhibitor ML349 had no substantial effect (*n* = 3). (D) ELISA results revealed that 2-BP treatment markedly reduced CCL20 expression levels in inflamed renal TECs, while ML349 treatment showed no substantial impact (*n* = 3). (E) A schematic illustration delineated the intervention protocol utilizing 2-BP in BSA nephritis mice. Following a 12-week disease induction period, mice assigned to the treatment cohort received intraperitoneal administration of 2-BP at a dosage of 40 mg/kg, delivered once per week for an additional 12 weeks. In parallel, control animals were given an equal volume of vehicle solution according to the same schedule, thereby ensuring consistency between experimental groups. (F to J) Representative images of Sirius Red staining and IF staining (α-SMA, vimentin, and ROR-γ, Cy3-labeled, red fluorescence; *n* = 6; scale bar, 20 μm) of serial kidney sections from BSA nephritis mice showed reduced renal fibrosis following 2-BP treatment. (K to N) In line with these histological improvements, 2-BP administration also markedly preserved renal function, as evidenced by lower levels of urinary protein (K), urinary protein-to-creatinine ratio (L), serum creatinine (M), and blood urea nitrogen (N) in the BSA nephritis mice (*n* = 6). (O to R) WB revealed that administration of 2-BP led to a marked reduction in the renal expression of ROR-γ, IL-17A, α-SMA, and vimentin in BSA nephritis mice. To ensure equal protein loading, β-actin and vinculin served as internal control proteins throughout the assays. The right panel presents semiquantitative analyses derived from the corresponding WB data, thereby providing quantitative support for the observed changes. Differences among 3 or more groups are assessed by one-way ANOVA. All results are expressed as mean ± SD. Statistical significance is denoted as follows: ns, not significant; *P < 0.05; **P < 0.01; ***P < 0.001; ****P < 0.0001. The schematic diagram in (E) is created using BioRender.com under a valid license.

Given that S-palmitoylation is a reversible lipid modification regulated by both PATs and APTs [23], we sought to comprehensively evaluate the pathogenic role of palmitoylation in IgAN-CKD by modulating both palmitoylation and depalmitoylation processes. Specifically, we inhibited ZDHHC14-mediated palmitoylation using 2-BP [26] and blocked APT2-mediated depalmitoylation using ML349 [41]. We first confirmed the direct effects of these interventions on TEAD4 itself. In inflamed pmTECs, the pan-PAT inhibitor 2-bromopalmitate (2-BP) markedly diminished TEAD4 palmitoylation levels, whereas the APT inhibitor ML349 enhanced this modification (Fig. 8A and B). This change in posttranslational status directly governed downstream chemokine expression; 2-BP treatment markedly reduced CCL20 levels, while ML349 treatment had the opposite effect (Fig. 8C and D). These findings suggest that inhibiting depalmitoylation exacerbates Th17 cell infiltration and promotes disease progression, while suppressing palmitoylation mitigates these effects.

To further evaluate the therapeutic potential of 2-BP in vivo, BSA nephritis mice were treated with 2-BP starting at week 12 post-induction. Mice received intraperitoneal injections of 40 mg/kg 2-BP once weekly until sacrifice at week 24 (Fig. 8E). Compared to the vehicle-treated group, 2-BP treatment markedly alleviated renal fibrosis, as evidenced by reduced α-smooth muscle actin (α-SMA) and vimentin-positive areas in the renal interstitium, and reduced infiltration of Th17 cells (ROR-γ-positive cells), as shown by IF staining (Fig. 8F to J). Additionally, compared to the vehicle-treated group, 2-BP administration led to a profound improvement in renal function, evidenced by marked reductions in 24-h proteinuria, urine protein-to-creatinine ratio, serum creatinine, and blood urea nitrogen (Fig. 8K to N). Consistent with this, the expression of key Th17-associated molecules, including ROR-γ and IL-17A, was also substantially suppressed (Fig. 8O to R). Taken together, these results demonstrate that pharmacological inhibition of PATs, including ZDHHC14, effectively disrupts the proinflammatory cascade, leading to reduced Th17 cell infiltration and amelioration of tubulointerstitial fibrosis. This validates palmitoylation as a promising therapeutic target for mitigating the progression of CKD.

## Discussion

**Fig. 9.**
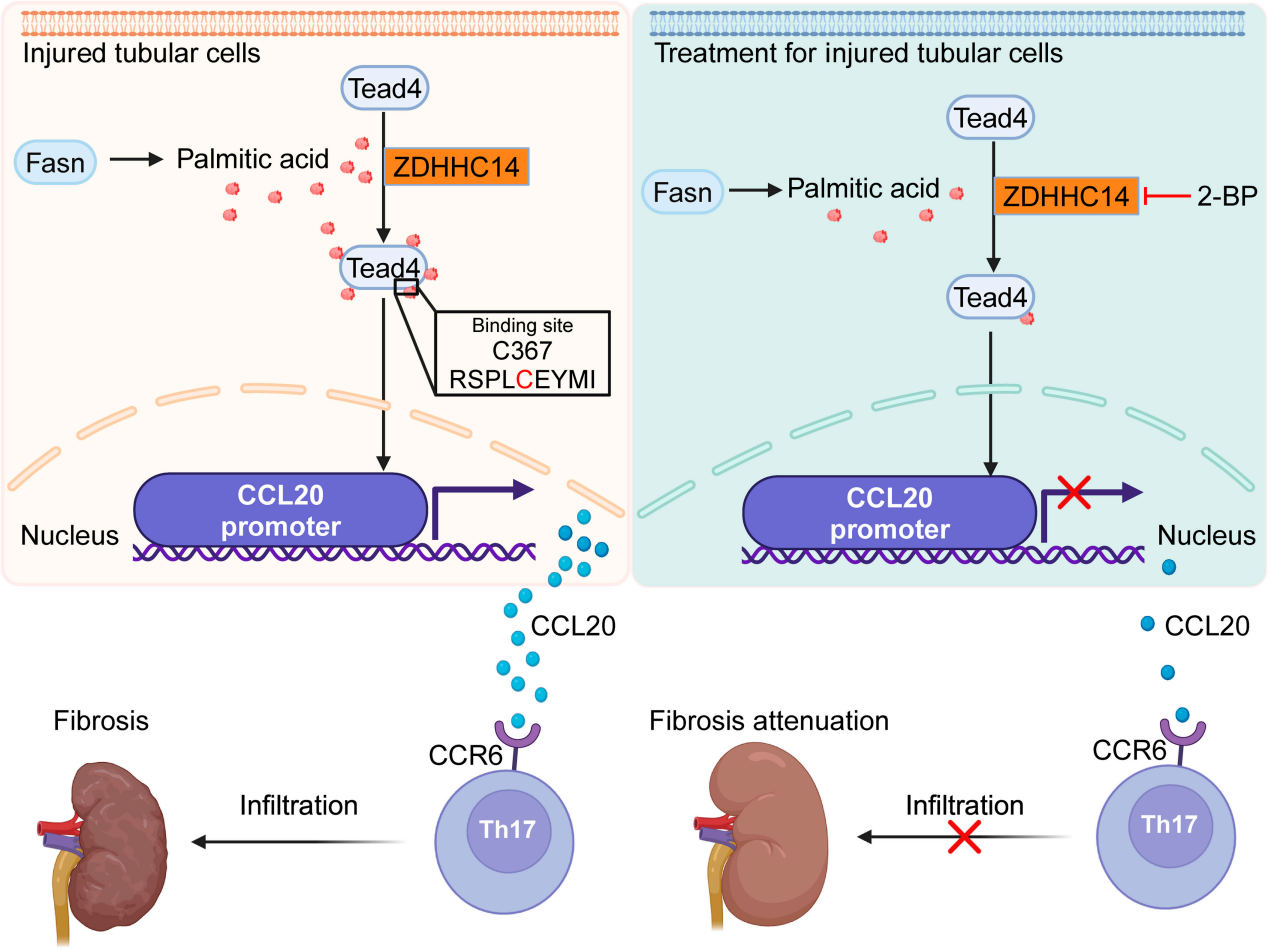
Schematic illustrating the mechanism by which ZDHHC14-mediated TEAD4 palmitoylation in inflamed renal TECs promotes renal fibrosis progression. Under inflammatory conditions, injured TECs up-regulate the chemokine CCL20 via the transcription factor TEAD4, driving Th17 cell recruitment. Crucially, TEAD4 activity is governed by palmitoylation rather than protein abundance. We identify ZDHHC14 as the key enzyme mediating TEAD4 palmitoylation. Pharmacological inhibition of ZDHHC14 (e.g., 2-BP) disrupts this axis, markedly reducing Th17 infiltration and attenuating disease progression, highlighting its therapeutic potential for CKD. The scheme is created using BioRender.com under a valid license.

This study is the first to demonstrate that ZDHHC14-mediated palmitoylation of TEAD4 promotes Th17 cell infiltration in immune glomerulonephritis. Our findings reveal that renal TECs, when subjected to inflammatory injury, produce elevated levels of the Th17 cell-recruiting chemokine CCL20. The expression of CCL20 is regulated by the transcription factor TEAD4 whose activity is enhanced following palmitoylation. This palmitoylation process is mediated by ZDHHC14 and relies on palmitic acid synthesized by FASN. Importantly, we discovered that inhibition of ZDHHC14 expression reduces TEAD4 palmitoylation, leading to a down-regulation of CCL20 expression and subsequently diminishing Th17 cell infiltration. These findings propose ZDHHC14 as a potential therapeutic target for renal fibrotic progression (Fig. 9).

Th17 cells, characterized by their secretion of IL-17, are widely recognized as a proinflammatory T cell subset [42–44]. These cells express the chemokine receptor CCR6, which specifically binds to the chemokine CCL20 [45–47]. Elevated levels of CCL20 have been reported in various tissues and diseases, where it facilitates Th17 cell infiltration [48,49]. However, the precise mechanisms underlying this process have remained unclear. In this study, using UCSC and JASPAR databases, we identified TEAD4 as a transcription factor for the CCL20 promoter and, for the first time, demonstrated that TEAD4 directly drives CCL20 expression. Our study establishes TEAD4 as a direct transcriptional activator of the CCL20 promoter, providing the first mechanistic evidence via dual-luciferase assays. This in vitro system, while confirming a direct link, may not reflect the full magnitude of effect seen in a native cellular context. This distinction, however, explains why the modest transcriptional activation observed translates into a more substantial increase in CCL20 protein, as quantified by ELISA. We propose that this is due to a “biological amplification” cascade within the cell. Collectively, our data form a complementary evidence chain: The luciferase assay demonstrates the regulation’s directness, while ELISA confirms its functional significance. Interestingly, we observed that TEAD4 expression levels were not elevated either in the renal tissues of disease models or in injured pmTECs. This suggests that under pathological or injury conditions, the transcriptional regulation of CCL20 by TEAD4 is likely attributed to enhanced TEAD4 activity rather than increased expression.

Previous studies have shown that the transcriptional activity of TEAD4 is regulated by palmitoylation, a PTM influenced by FASN expression [29,30,32,50–52]. FASN, through its up-regulation, promotes the synthesis of palmitic acid esters, which in turn enhance TEAD4 palmitoylation [29]. Consistent with these findings, our study demonstrates that FASN-mediated palmitoylation of TEAD4 plays a critical role in amplifying CCL20 expression, thereby promoting Th17 cell infiltration. Additionally, single-cell transcriptomic analysis of kidney tissues from IgAN patients revealed substantially elevated expression of lipid metabolism-related genes, including FASN. This finding was further validated in the renal tissues of BSA nephritis mice. The aberrant expression of FASN is closely associated with lipid metabolism disorders, which are key contributors to the progression of CKD, regardless of the underlying primary renal disease [53,54]. Dysregulated fatty acid metabolism, characterized by lipid accumulation and impaired fatty acid oxidation, leads to mitochondrial dysfunction, energy metabolism imbalance, and TEC injury [55–58]. These alterations in metabolic pathways give rise to the production of proinflammatory molecules, promote the infiltration of immune cell populations, and trigger fibroblast activation, thereby contributing to the progression and worsening of renal fibrosis [59,60]. Lipid metabolism abnormalities have been shown to synergistically contribute to the progression of immune-inflammatory kidney diseases [61], including the pathogenic effects of protein lipidation modifications [62]. Among PTMs, palmitoylation plays a particularly notable role in renal physiology [26,63]. In this study, we identified ZDHHC14 as the PAT responsible for mediating TEAD4 palmitoylation. Importantly, this pathological process can be reversed by 2-BP, resulting in reduced CCL20 expression and diminished Th17 cell infiltration.

Interestingly, the ZDHHC family comprises 24 members, each exhibiting distinct expression patterns and regulatory roles in different pathological contexts. In our study, we observed that not all ZDHHC isoforms were uniformly expressed in the renal tissues of the disease model. For isoforms with low expression, their potential pathogenic roles may be masked or mediated through alternative regulatory mechanisms. For instance, previous research has demonstrated that ZDHHC9 is down-regulated in both CKD patients and acute kidney injury (AKI)-CKD mouse models, where it exerts a protective effect on the kidney by mediating β-catenin palmitoylation, promoting its ubiquitination and degradation [26]. In a similar context, recent findings demonstrate a substantial increase in ZDHHC18 expression within murine models of renal fibrosis generated by either unilateral ureteral ligation or folic acid treatment. Its profibrotic effects are primarily mediated via the regulation of harvey rat sarcoma (HRAS) palmitoylation, a critical PTM known to drive fibrotic signaling pathways [64]. Nevertheless, within the confines of our experimental system, no substantial changes were observed in the abundance of either ZDHHC9 or ZDHHC18 transcripts. Instead, the predominant pathogenic mechanism appeared to be driven by the pronounced up-regulation of ZDHHC14, which facilitated TEAD4 palmitoylation, thereby enhancing its transcriptional activity. These findings underscore the context-dependent roles of ZDHHC isoforms, suggesting that their pathogenic or protective effects are likely to vary depending on specific disease mechanisms. Further investigations are warranted to elucidate the precise contributions of individual ZDHHC isoforms in different pathological conditions.

While this study unveils a novel pathogenic pathway, we acknowledge several limitations that also chart a course for future investigations. First, the number of human kidney tissue samples analyzed was limited. Although these samples provided crucial clinical relevance for our mechanistic findings, confirming the up-regulation of IL-17A and ZDHHC14 in IgAN kidneys, validation in larger, multi-center, and independent patient cohorts with diverse disease stages and pathological features is essential to establish this pathway as a robust therapeutic target. Second, at the molecular level, the precise structural basis for the ZDHHC14-TEAD4 interaction remains to be elucidated. In this study, we employed the broad-spectrum inhibitor 2-bromopalmitate (2-BP) as a proof-of-concept tool, demonstrating the therapeutic potential of blocking this pathway. However, we fully acknowledge the inherent limitations of this pan-PAT inhibitor. Beyond its lack of specificity across the ZDHHC family, 2-BP, as a palmitic acid analog, can interfere with broader cellular fatty acid metabolism, raising concerns about potential off-target effects and toxicity that constrain its utility as a direct therapeutic agent [65,66]. This limitation underscores the critical need to develop and validate highly selective TEAD4 palmitoylation inhibitors. Such targeted agents would not only offer greater mechanistic precision but also represent a more viable path toward a safer and more effective clinical intervention. Nevertheless, the present study provides a novel and compelling framework linking lipid PTM to immune-driven tubulointerstitial injury in IgAN, paving the way for these exciting future investigations into a promising therapeutic pathway.

In conclusion, this study uncovers a previously unrecognized pathway underlying the advancement of IgAN toward CKD, whereby TECs subjected to inflammatory injury facilitate enhanced CCL20 transcription via ZDHHC14-dependent palmitoylation of TEAD4. This, in turn, recruits proinflammatory Th17 cells, which activate fibroblasts to secrete extracellular matrix, thereby accelerating disease progression. Importantly, inhibiting TEAD4 palmitoylation reduces Th17 cell recruitment and alleviates the fibrotic phenotype. These results provide new insights into the inflammatory and fibrotic processes in IgAN-CKD and suggest potential therapeutic strategies targeting TEC injury and immune dysregulation. Future studies will aim to refine these findings and explore their translational potential in clinical settings.

## Materials and Methods

### Human study and ethical approval

A cohort of 19 adult patients diagnosed with IgAN and an equal number of healthy controls, matched for gender and age, were recruited for serum sample collection. Additionally, renal tissue samples were procured from 3 patients during the course of the study. Normal kidney samples were collected from patients undergoing nephrectomy for renal carcinoma. This study was conducted following ethical guidelines and was approved by the Ethics Committee (approval number: S2022-388-01). In compliance with both federal and institutional requirements, informed consent was secured from every participant prior to enrollment. Baseline demographic and clinical features are detailed in Tables S1 and S2.

### Animal model and treatment

Balb/c mice, sourced from Cyagen Biosciences (Suzhou, China), were housed under specific pathogen-free conditions with ad libitum access to food and water, a 12-h light/dark cycle, and a constant temperature of 25 °C. All experimental protocols involving animals strictly complied with established welfare regulations and received authorization from the relevant Ethics Committee (approval no. TACU24-FY069). BSA nephritis mice were generated following previously published methodologies [67].

In the treatment cohort, beginning at the 12th week, mice received intraperitoneal injections of 2-BP (4 mg/kg; Sigma, USA) once per week, continuing through week 24 [26]. Animals subjected to this regimen were euthanized at week 24 for subsequent analyses.

### WB and Co-IP analysis

For WB, tissues or cells were lysed in radioimmunoprecipitation assay lysis buffer (RIPA; Beyotime, China) supplemented with 1 μM phenylmethylsulfonyl fluoride (PMSF; Beyotime, China). Equal amounts of protein (20 μg per sample) were loaded and analyzed. Detailed antibody information is provided in Table S3.

For Co-IP, cells were lysed in NP-40 buffer (Beyotime, China). Eluted proteins (100 μg per sample) were analyzed by WB. Input samples were loaded at 20 μg per lane. Antibody details are listed in Table 3.

### ABE and Click-iT assays for protein palmitoylation

For the ABE assay, cells were lysed in NP-40 buffer, and 500 μg of total protein was incubated with antibody-conjugated magnetic beads (ThermoFisher, USA) at 4 °C for 4 h. Beads were washed, free thiols were blocked, and proteins were reduced and labeled with hydroxylamine. Eluted (50 to 100 μg) and input samples (20 μg) were analyzed by WB.

For the Click-iT assay, cells were treated with azide-modified palmitic acid (ThermoFisher, USA) and incubated at 37 °C for 6 h. After lysis in NP-40 buffer, azide-labeled proteins were conjugated with biotin-alkyne through copper-catalyzed click chemistry. Complexes were enriched using streptavidin beads, washed, and analyzed by WB. Antibody information is provided in Table S3.

### Histological analysis

Paraffin sections (2 μm thick) were stained with PAS, Masson trichrome, or Sirius Red, or subjected to IHC staining. Frozen sections (4 μm thick) were used for IF staining [68].

### In vitro Th17 cell migration assay

The Th17 cell migration assay utilized a transwell coculture system. LPS-stimulated pmTECs were cultured in the lower chamber, with differentiated Th17 cells seeded in the upper chamber of a 5-μm transwell insert. After 24 h, migrated live cells in the lower chamber were stained with calcein AM and imaged by confocal microscopy. Cell migration was quantified by averaging counts from 10 random fields per sample.

### In vitro coculture of renal fibroblasts and Th17 cells

Primary renal fibroblasts were plated in 6-well dishes and permitted to attach for a period of 6 h. Subsequently, a 0.4-μm transwell insert (Corning, USA) was positioned above the fibroblast layer, after which differentiated Th17 cells were introduced into the upper compartment. The coculture system was maintained at 37 °C in a humidified atmosphere with 5% CO₂ for 24 h. After coculture, fibroblasts in the lower chamber were collected for further analysis.

### Enzyme-linked immunosorbent assay

Human serum IL-17 and CCL20 levels were measured using ELISA kits (R&D Systems, USA), with absorbance recorded at 450 nm and background corrected at 540 nm. All samples were analyzed in triplicate for accuracy and reproducibility.

### Dual-luciferase reporter assay

To confirm the binding of the transcription factor TEAD4 to the CCL20 promoter, a dual-luciferase reporter assay was performed (Promega, USA). The CCL20 promoter region was cloned into the pGL3-Basic vector, with pRL-TK serving as an internal control. HEK293 cells or pmTECs were cotransfected with these constructs and TEAD4 overexpression plasmids (human or mouse) or an empty vector. Firefly and renilla luciferase activities were sequentially measured at 460 nm, and firefly activity was normalized to renilla activity to calculate relative luciferase activity. Predicted TEAD4 binding sites on the CCL20 promoter are detailed in Table S4.

### RT-qPCR assay

Total RNA was extracted from cells or tissues using TRIzol reagent (Invitrogen). The relative mRNA expression levels were subsequently quantified via RT-qPCR, performed on an Applied Biosystems 7500 Real-Time PCR System (Applied Biosystems, Foster City, CA). All primers used for this study are detailed in Table S5.

### ChIP and DNA pull-down assays

ChIP assays were performed using the ChIP Assay Kit (Beyotime, P2083S) in strict accordance with the manufacturer’s protocol. Briefly, cells were subjected to formaldehyde fixation to cross-link proteins to DNA. The cross-linked chromatin was then sheared into fragments via micrococcal nuclease (MNase) digestion and subsequently immunoprecipitated using an antibody specific to the protein of interest. The enrichment of specific DNA sequences was quantified by RT-qPCR and visualized on an agarose gel stained with SYBR Green. The primers designed to amplify the murine Ccl20 promoter region were: 5′-GCCTCTCGTACATACAGACGC-3′ (forward) and 5′-CCAGTTCTGCTTTGGATCAGC-3′ (reverse).

For the DNA pull-down experiments, conducted with the Pull-Down Assay Kit (Beyotime, P0637S), a biotin-labeled double-stranded DNA probe corresponding to the Ccl20 promoter was synthesized and immobilized on streptavidin-coated magnetic beads. These DNA probe-conjugated beads were then incubated with nuclear protein extracts to capture specific DNA-binding proteins. Following rigorous washing steps to eliminate nonspecifically bound molecules, the captured proteins were eluted. The presence and relative abundance of TEAD4 in the eluate were subsequently determined by WB analysis.

### Statistical analysis

Statistical analyses were conducted using GraphPad Prism version 9.0. For comparisons between 2 groups, Student’s *t* test was applied, whereas one-way analysis of variance (ANOVA) with Tukey’s post hoc test was employed when evaluating 3 or more groups. Additionally, Pearson’s correlation analysis was used to assess the relationship between 2 variables. Data are presented as mean ± SD, and a *P* value of less than 0.05 was deemed statistically significant.

## Data Availability

The data underlying this article are available in the article and the Supplementary Materials. All of the raw RNA-sequencing data discussed in this publication have been deposited in NCBI Gene Expression Omnibus with the following IDs: GSE287800 (https://www.ncbi.nlm.nih.gov/geo/query/acc.cgi?acc=GSE287800) and GSE287962 (https://www.ncbi.nlm.nih.gov/geo/query/acc.cgi?acc=GSE287962). All of the raw metabolomics data discussed in this publication have been deposited in the OMIX in the National Genomics Data Center, China National Center for Bioinformation/Beijing Institute of Genomics, Chinese Academy of Sciences with the following ID: OMIX008796 (https://ngdc.cncb.ac.cn/omix/).
