# Supplementary material for: ZDHHC14-Mediated TEAD4 Palmitoylation Drives Th17 Cell Recruitment in Renal Immunopathology
Source: Research (Wash D C). 2025 Oct 16;8:0954. doi: 10.34133/research.0954 (PMC12529098; doi:10.34133/research.0954)
Supplement: Supplementary 1 — Figs. S1 to S9 Tables S1 to S5 [file research.0954.f1.docx]

**Supplmentary Materials**

**Supplmentary methods**

**Primary Mouse Tubular Epithelial Cell (pmTEC) Isolation**

Kidney tissues from mice were minced and digested with 2 mg/ml collagenase I at 37°C with gentle shaking for 30 minutes. The digested mixture was neutralized with Dulbecco's modified Eagle's medium-F12 (DMEM-F12) containing 10% fetal bovine serum (FBS). After centrifugation, the cell suspension was passed through 100 μm and 40 μm sieves sequentially, and red blood cells were lysed using red cell lysis buffer. The cells were washed three times with PBS and cultured in DMEM-F12 medium supplemented with 10% FBS, 1% penicillin-streptomycin, 1× insulin-transferrin-selenium, and 20 ng/ml murine epidermal growth factor (EGF). For small interfering RNA (siRNA) transfection, pmTECs were transfected with siRNA and transfection reagent when cell confluence reached 60%. After 48 hours, cells were collected for further analysis. For Zdhhc14 overexpression, a bacterial plasmid was constructed, and cells were transfected at 80% confluence using a transfection reagent. Cells were harvested 36 hours post-transfection. Specific sequence information is provided in Table S3.

**Renal Fibroblast Isolation**

Mouse kidney tissues were digested in a solution containing PBS, 0.25% trypsin, and 0.2% collagenase IV at 37°C with gentle shaking for 30 minutes. The digested mixture was neutralized with Fibroblast Medium Complete (ScienCell, 2301), filtered through a 70 μm sieve, and red blood cells were lysed using red cell lysis buffer. The cells were washed three times with PBS and cultured in Fibroblast Medium Complete.

**CD4+ T Cell Isolation**

Mouse spleens were minced and ground into single-cell suspensions, which were passed through a 70 μm sieve. Red blood cells were lysed using red cell lysis buffer, and the remaining cells were washed three times with PBS. CD4+ T cells were isolated using magnetic beads and resuspended in RPMI 1640 medium supplemented with GlutaMAX and 10% FBS for culture.

**In Vitro Th17 Cell Differentiation**

To induce pathogenic Th17 cell differentiation, 24-well plates were pre-coated with 10 μg/ml anti-CD3 and 10 μg/ml anti-CD28 antibodies. Plates were incubated at 37°C for 4 hours and subsequently stored at 4°C until use. Before cell seeding, the plates were washed three times with PBS to remove unbound antibodies. CD4+ T cells were isolated and resuspended in 500 μl of serum-free medium supplemented with the following inducers: 20 ng/ml IL-1β, 40 ng/ml IL-6, 50 ng/ml IL-23, and 20 μg/ml anti-IFN-γ. The cell suspension was added to the prepared 24-well plates, and cells were cultured for 5 days to induce differentiation into Th17 cells. After induction, cells were collected for subsequent experiments [1].

**Cell Culture and Treatment**

Human Embryonic Kidney (HEK293T) Cells were purchased from iCell Bioscience (Shanghai, China) and cultured in high-glucose DMEM (Gibco) supplemented with 10% FBS.

**Cell Proliferation Assay**

For the cell-counting-kit-8 (CCK-8) assay, 1×10⁴ pmTEC cells were seeded per well in a 96-well plate. Lipopolysaccharide (LPS) was added at concentrations of 0, 50, 100, 500, 1000, 5000, 10000, and 50000 ng/ml. Cell proliferation was assessed using the CCK-8 kit according to the manufacturer’s instructions.

**Metabolomics**

Metabolite extraction was performed using 25 mg of tissue homogenized with beads in 500 μL of extraction solution (methanol: acetonitrile: water = 2:2:1, v/v) containing isotopically labeled internal standards. The mixture was vortexed for 30 seconds, homogenized at 35 Hz for 4 minutes, and sonicated in an ice-water bath for 5 minutes, repeated three times. After incubation at -40°C for 1 hour, samples were centrifuged at 12,000 rpm (13,800 × g) at 4°C for 15 minutes. The supernatant was collected for analysis, and a pooled quality control (QC) sample was prepared by mixing equal volumes of all supernatants. Polar metabolites were analyzed using a Vanquish ultra-high-performance liquid chromatography (UHPLC) system (Thermo Fisher Scientific) equipped with a Waters ACQUITY UPLC BEH Amide column (2.1 mm × 50 mm, 1.7 μm). The mobile phase consisted of 25 mmol/L ammonium acetate and 25 mmol/L ammonia water (phase A) and acetonitrile (phase B). The sample tray was maintained at 4°C, and the injection volume was 2 μL. Mass spectrometry data were acquired using an Orbitrap Exploris 120 mass spectrometer under Xcalibur software (version 4.4, Thermo). Key parameters included a sheath gas flow rate of 50 Arb, auxiliary gas flow rate of 15 Arb, capillary temperature of 320°C, full MS resolution of 60,000, MS/MS resolution of 15,000, collision energy of 20/30/40, and spray voltage of 3.8 kV (positive mode) or -3.4 kV (negative mode) [2]. Raw data were converted to mzXML format using ProteoWizard and processed with an in-house R-based program utilizing XCMS for peak detection, alignment, and integration. Normalized values were calculated relative to the control group, and heatmaps of fatty acid expression patterns were generated using the "pheatmap" package in R4.3.2. Palmitic acid levels were compared between groups using t-tests, and box plots were created with the "ggplot2" package to visualize differences.

**Transcriptomics Analysis**

Total RNA (50 μg) was extracted from fibroblasts and kidney tissues of IgAN and control mice using standard protocols. Library preparation was performed with the VAHTS Universal V6 RNA-seq Library Prep Kit for Illumina®, and cDNA library quality was assessed using an Agilent 4200 bioanalyzer. Sequencing was conducted on an Illumina NovaSeq 6000 platform following the manufacturer’s instructions. Raw sequencing reads were filtered for quality using Seqtk and aligned to the reference genome with Hisat2 (version 2.0.4) [3]. Gene fragment counts were quantified using StringTie (v1.3.3b), and normalization was performed using the trimmed mean of M values (TMM) method. Differentially expressed genes (DEGs) were identified using edgeR software, applying a False Discovery Rate (FDR) threshold of Q < 0.05 and a fold-change >2 [4-6]. Functional enrichment analysis of DEGs was performed using the "clusterProfiler" package in R4.3.2, focusing on Kyoto Encyclopedia of Genes and Genomes (KEGG) pathways. The top 30 enriched pathways were visualized with the "ggplot2" package, with statistical significance set at P < 0.05. DEG expression patterns were further analyzed using heatmaps generated with the "pheatmap" package. Correlation analysis between TEAD4 and other genes was conducted using the "correlation" package in R4.3.2, calculating Pearson correlation coefficients and corresponding p-values. Results were visualized with bubble plots and scatter plots to illustrate the distribution and significance of correlations [7-9].

**Table S1. Basic characteristics of IgAN patients (blood samples)**

| ID | Age | Gender | BUN  (mmol/L) | Scr  (ummol/L) | 24hUTP  (g) | eGFR-EPI | Oxford classification |
| --- | --- | --- | --- | --- | --- | --- | --- |
| Patient 1 | 60 | M | 6.79 | 124.4 | 5.1148 | 56.11 | M1E0S1T1C1 |
| Patient 2 | 25 | F | 5.44 | 158.1 | 0.9219 | 40.73 | M0E0S1T2C0 |
| Patient 3 | 48 | M | 6.53 | 157.7 | 0.2619 | 46.49 | M030S1T2C0 |
| Patient 4 | 33 | M | 10.6 | 232.5 | 4.73 | 32.29 | M1E1S1T0C1 |
| Patient 5 | 31 | M | 9,1 | 179.1 | 2.8706 | 44.91 | M1E0S1T1C0 |
| Patient 6 | 43 | M | 8,35 | 167.3 | 2.9589 | 44.82 | M1E0S1T1C0 |
| Patient 7 | 35 | M | 9.78 | 231.2 | 2.0307 | 32.06 | M0E1S1T1C1 |
| Patient 8 | 53 | M | 7.73 | 149.2 | 2.208 | 47.99 | M1E0S1T1C1 |
| Patient 9 | 61 | F | 10.12 | 141.2 | 1.7336 | 36.26 | M0E1S1T1C1 |
| Patient 10 | 34 | M | 8.02 | 154.1 | 1.0208 | 52.74 | M0E1S1T0C0 |
| Patient 11 | 49 | F | 10.27 | 135.3 | 1.463 | 41.54 | M0E1S1T1C0 |
| Patient 12 | 26 | F | 7.91 | 159 | 0.8854 | 40.16 | M0E0S1T1C0 |
| Patient 13 | 37 | M | 7.65 | 180.5 | 0.2952 | 42.65 | M0E0S1T0C0 |
| Patient 14 | 42 | M | 7.5 | 182.7 | 0.2907 | 40.58 | M1E0S1T1C0 |
| Patient 15 | 52 | M | 7.91 | 141.8 | 1.372 | 51.76 | M0E0S1T1C0 |
| Patient 16 | 69 | M | 4.74 | 137.6 | 2.4472 | 47.3 | M0E0S1T2C1 |
| Patient 17 | 54 | F | 6.16 | 123.3 | 2.5608 | 44.88 | M0E0S1T0C1 |
| Patient 18 | 55 | M | 10.46 | 175.5 | 3.1837 | 38.88 | M0E0S1T1C1 |
| Patient 19 | 66 | F | 9.86 | 124.1 | 1.7864 | 40.93 | M0E0S1T1C0 |

**Table S2. Basic characteristics of IgAN patients (kidney biopsy samples)**

| ID | Age | Gender | BUN  (mmol/L) | Scr  (ummol/L) | 24hUTP  (g) | eGFR-EPI | Oxford classification |
| --- | --- | --- | --- | --- | --- | --- | --- |
| Patient-1 | 36 | M | 6.31 | 111.7 | 1.116 | 76.76 | M1E0S1T1C1 |
| Patient-2 | 47 | M | 7.4 | 120.9 | 2.8377 | 64.57 | M1E0S1T1C1 |
| Patient-3 | 35 | M | 6.01 | 131.6 | 0.819 | 63.39 | M0E0S1T1C0 |

**Table S3. Antibody information for western blotting, co-immunoprecipitation, ABE&Click-iT assay, immunohistochemistry and immunofluorescence staining**

| Antibody | Source | Identifier | Concentration |
| --- | --- | --- | --- |
| **Western blotting** | | | |
| α-SMA | abcam | ab7817 | 1 : 3000 |
| Vinculin | proteintech | 66305-1 | 1 : 20000 |
| Vimentin | abcam | ab92547 | 1 : 1000 |
| IL-17A | abcam | ab79056 | 1 : 2000 |
| β-actin | proteintech | 66009-1 | 1 : 20000 |
| Bmp2 | abcam | ab284387 | 1 : 1000 |
| p-Smad1/5/9 | CST | 13820 | 1 : 1000 |
| Smad1 | Santa Cruz | sc-7965 | 1 : 1000 |
| p-Smad2/3 | CST | 8828 | 1 : 1000 |
| Smad2/3 | CST | 8685 | 1 : 1000 |
| Collagen I | proteintech | 14695-1-AP | 1 : 1000 |
| Tead4 | Santa Cruz | sc-390578 | 1 : 500 |
| Fasn | proteintech | 66591-1-Ig | 1 : 5000 |
| Zdhhc14 | biorbyt | orb422936 | 1: 2000 |
| ROR gamma | abcam | ab207082 | 1: 2000 |
| Goat Anti-Rabbit IgG(H+L) | Beyotime | P0948 | 1: 2000 |
| Goat Anti-Mouse IgG(H+L) | Beyotime | P0946 | 1: 2000 |
| **Co-immunoprecipitation** | | | |
| Tead4 | Santa Cruz | sc-390578 | 1 : 500 |
| Zdhhc14 | biorbyt | orb422936 | 1 : 2000 |
| Mouse Anti-Rabbit IgG (Light-Chain Specific) | CST | 93702 | 1 : 1000 |
| Rabbit Anti-Mouse IgG (Light Chain Specific) | CST | 58802 | 1 : 1000 |
| **ABE&Click-iT assays** | | | |
| Tead4 | Santa Cruz | sc-390578 | 1 : 500 |
| Vinculin | proteintech | 66305-1 | 1 : 20000 |
| Zdhhc14 | biorbyt | orb422936 | 1 : 2000 |
| Zdhhc1 | Invitrogen | PA5-113194 | 1 : 2000 |
| Zdhhc21 | Invitrogen | PA5-25096 | 1 : 1000 |
| β-actin | proteintech | 66009-1 | 1 : 20000 |
| Goat Anti-Rabbit IgG(H+L) | Beyotime | P0948 | 1 : 2000 |
| Goat Anti-Mouse IgG(H+L) | Beyotime | P0946 | 1 : 2000 |
| **Immunohistochemistry** | | | |
| IL-17A | abcam | ab79056 | 1 : 400 |
| ROR gamma | abcam | ab207082 | 1 : 3000 |
| Zdhhc14 | biorbyt | orb422936 | 1 : 500 |
| **Immunofluorescence** | | | |
| IgA-FITC | Novus Biologicals | NB7503 | 1 : 100 |
| Collagen I | abcam | Ab270993 | 1 : 2000 |
| Fasn | proteintech | 66591-1-Ig | 1 : 400 |
| LTL | VECTOR | FL-1321 | 1 : 200 |
| α-SMA | abcam | ab7817 | 1 : 1000 |
| Vimentin | abcam | ab92547 | 1 : 500 |
| ROR gamma | abcam | ab207082 | 1 : 500 |
| Goat Anti-Rabbit(Cy3) | Beyotime | A0516 | 1 : 400 |
| Goat Anti-Mouse(Cy3) | Beyotime | A0521 | 1 : 400 |

**Table S4. Probable binding sites between transcription factor TEDA4 and CCL20 promoter predicted by JASPAR**

| **Name** | **Score** | **Relative score** | **Start** | **End** | **Strand** | **Predicted sequence** |
| --- | --- | --- | --- | --- | --- | --- |
| MA0809.3.TEAD4 | 14.6 | 1.00 | 1042 | 1049 | - | ACATTCCA |
| MA0809.2.TEAD4 | 14.8 | 0.97 | 1040 | 1051 | - | GAACATTCCAGA |
| MA0809.1.TEAD4 | 11.8 | 0.97 | 1041 | 1050 | - | AACATTCCAG |
| MA0809.3.TEAD4 | 10.7 | 0.92 | 1353 | 1360 | + | ACATTCCC |
| MA0809.1.TEAD4 | 9.3 | 0.92 | 1179 | 1188 | - | TAAATTCTTT |
| MA0809.1.TEAD4 | 9.2 | 0.92 | 1352 | 1361 | + | TACATTCCCA |
| MA0809.1.TEAD4 | 8.4 | 0.90 | 228 | 237 | + | AAAATTCTGT |
| MA0809.2.TEAD4 | 10.0 | 0.88 | 1351 | 1362 | + | GTACATTCCCAA |

**Table S5. Gene-specific siRNA oligo sequence mentioned in the study**

| **Gene** | **Sense** | **Antisense** |
| --- | --- | --- |
| siNC | 5’-UUCUCCGAACGUGUCACGUTT-3’ | 5’-ACGUGACACGUUCGGAGAATT-3’ |
| siFasn | 5’-GGAUCAACCUGCUCCUGAATT-3’ | 5’-UUCAGGAGCAGGUUGAUCCTT-3’ |
| siZdhhc14 | 5’-GCCAGACCGUGAAACUAAATT-3’ | 5’-UUUAGUUUCACGGUCUGGCTT-3’ |
| siTead4 | 5’-GCCACUAUUUGUACCGCAUTT-3’ | 5’-AUGCGGUACAAAUAGUGGCTT-3’ |
| siZdhhc1 | 5’-GCAAGGAGAUGGAGUUCUACA-3’ | 5’-UAGAACUCCAUCUCCUUGCGG-3’ |
| siZdhhc21 | 5’-GGUUAUACAAUAUUGUUAUAA -3’ | 5’-AUAACAAUAUUGUAUAACCAG-3’ |

**Table S5. The primers of RT-qPCR mentioned in the study**

| **Gene** | **Forward Primer** | **Reverse Primer** |
| --- | --- | --- |
| Zdhhc1 | 5’-UUCUCCGAACGUGUCACGUTT-3’ | 5’-ACGUGACACGUUCGGAGAATT-3’ |
| Zdhhc2 | 5’-GGAUCAACCUGCUCCUGAATT-3’ | 5’-UUCAGGAGCAGGUUGAUCCTT-3’ |
| Zdhhc3 | 5’-GCCAGACCGUGAAACUAAATT-3’ | 5’-UUUAGUUUCACGGUCUGGCTT-3’ |
| Zdhhc4 | 5’-TGATTTGTGTTGTCCTGATCTGC-3’ | 5’-GGAGGCACTGCGGGATTAC-3’ |
| Zdhhc5 | 5’-CCCAGGAATTTTTCCCCGAG-3’ | 5’-CACACCATTTCATTCGCACCT-3’ |
| Zdhhc6 | 5’-CATAGCCCTGGGTGTTATAGCA-3’ | 5’-CCTGAGACTTTTCCGGTTTCC-3’ |
| Zdhhc7 | 5’-TCGTCTATGCAGACTTCGTGG-3’ | 5’-GGGTCAGTGAGCATGGTTCT-3’ |
| Zdhhc8 | 5’-AGGTCCGCATGAAGTGGTG-3’ | 5’-GGCGTCCAATGCAGTTGTTG-3’ |
| Zdhhc9/10 | 5’-TTCTTTGCCTTCGAGTGTCG-3’ | 5’-CTGCTTCATCTGGTAGTGCTC-3’ |
| Zdhhc11 | 5’-CTGACACCAATGTCCGACTCA-3’ | 5’-CGCGGTAACCTCACACAGG-3’ |
| Zdhhc12 | 5’-ACCCCGGCTATGTGACTACTC-3’ | 5’-TGCGTTCACCCACACAGTTC-3’ |
| Zdhhc13 | 5’-ACTGGGCTGCCATTAACAACA-3’ | 5’-GCAAATGCCCTTGTCGGATG-3’ |
| Zdhhc14 | 5’-CAGAGCACCAAATTCGTTTTGC-3’ | 5’-GGGCATAATGTCTGAGAGTGTG-3’ |
| Zdhhc15 | 5’-GCCATCTAATCAAGCCAGACC-3’ | 5’-ACAGGCAGTAGAGAACGGAATAA-3’ |
| Zdhhc16 | 5’-TGGCGTTATGGCAAGGTTTG-3’ | 5’-CAGCGGATCACATTGTCCAC-3’ |
| Zdhhc17 | 5’-GGAGGGATTTAACACCAAGATGG-3’ | 5’-AGTTTTCCGACCAAGAGGTTCT-3’ |
| Zdhhc18 | 5’-TCGTCTTCGACTGTCCCTACC-3’ | 5’-GGGTCCGTGAAACTGGTCTG-3’ |
| Zdhhc19 | 5’-CCTGGTATCTTACATCGAGGCT-3’ | 5’-GAAGTCCTCCACGCAAATGTT-3’ |
| Zdhhc20 | 5’-GGAAAGACCGTTGTTTACCTTGT-3’ | 5’-ACTCCTTCTCATAACGCTCCTT-3’ |
| Zdhhc21 | 5’-ATGGGTCTTCGGATTCACTTTG-3’ | 5’-GCCCTCACTAAGGCAACCAG-3’ |
| Zdhhc22 | 5’-CGGCTGCTCAACGTGGTAG-3’ | 5’-CCAGGACGTAATTGCCCAGG-3’ |
| Zdhhc23 | 5’-GGCTGCCTGTTTGTGTGATTG-3’ | 5’-CCGTGATTCTTTCGCAAGTCTC-3’ |
| Zdhhc24 | 5’-GGATGCCACTGGTACTCACC-3’ | 5’-CCCAGCACCATCACGTAGG-3’ |
| 18s | 5’-GTAACCCGTTGAACCCCATT-3’ | 5’-CCATCCAACGGTAGTAGCG-3’ |


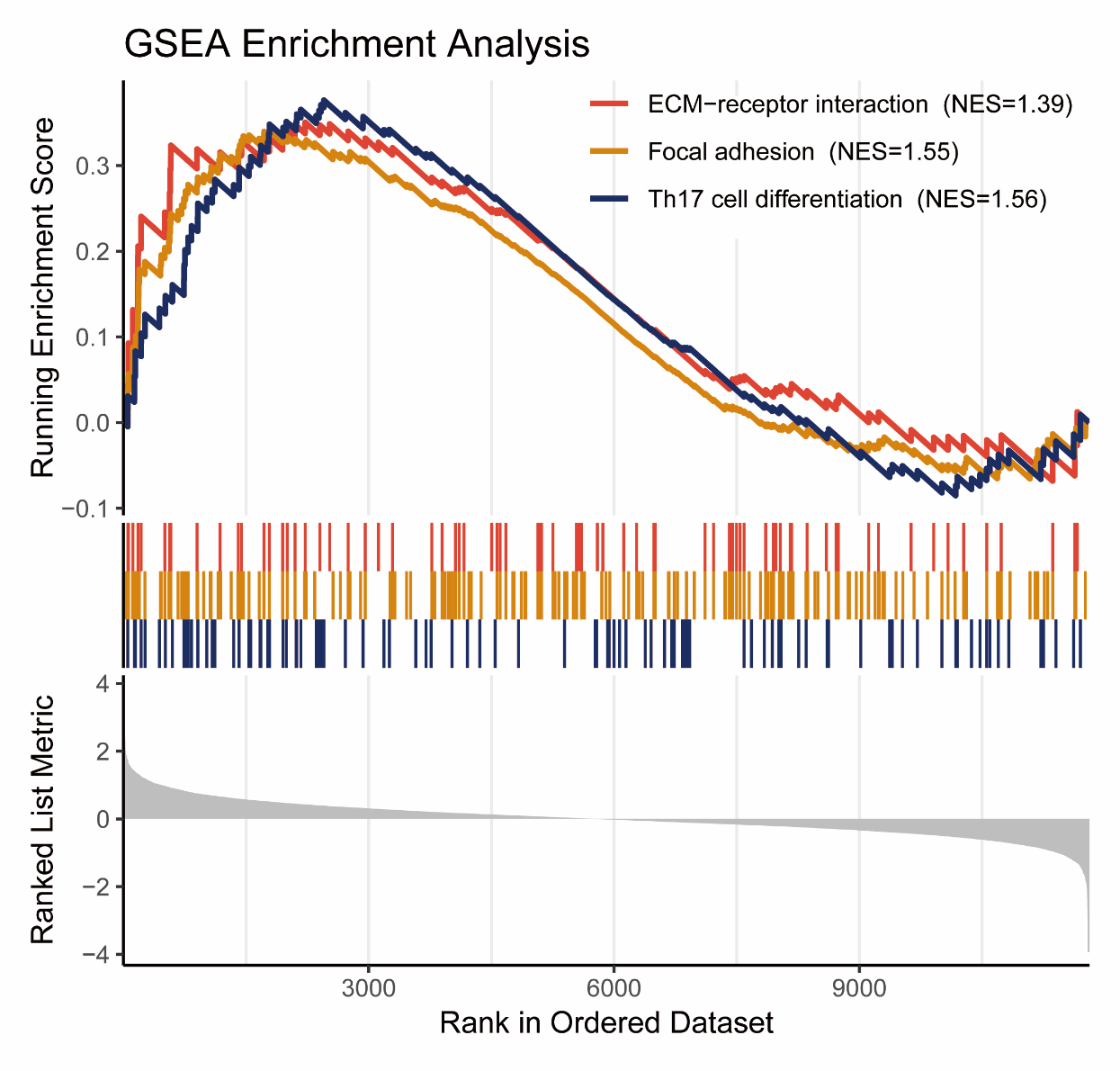


**Fig. S1.** Gene Set Enrichment Analysis (GSEA) of the renal transcriptome from IgAN patients (GEO: GSE37463) revealed the concurrent and significant upregulation of both the Th17 cell differentiation and fibrosis-associated pathways.


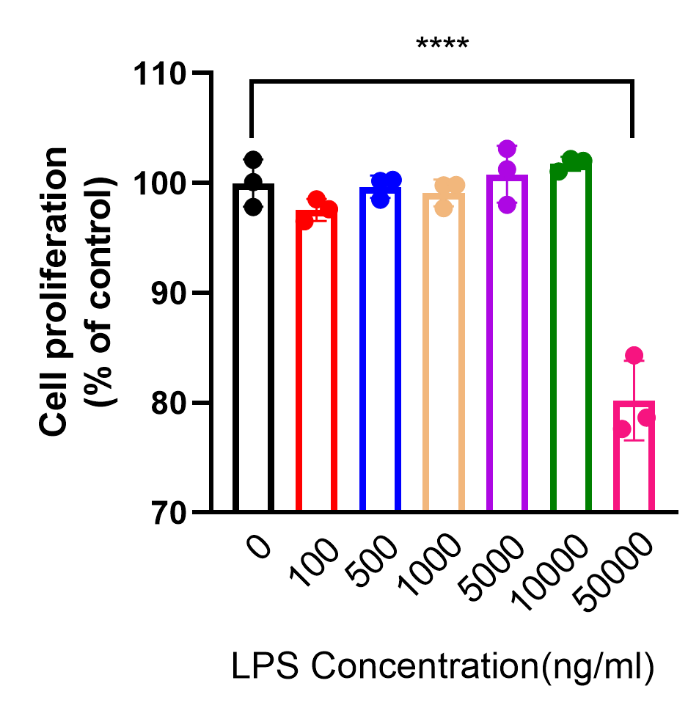


**Fig. S2.** CCK8 assay to determine the optimal concentration of lipopolysaccharide (LPS) for stimulating primary mouse tubular epithelial cells (pmTEC). A series of LPS concentrations—0, 100, 500, 1,000, 5,000, 10,000, and 50,000 ng/mL—was applied to the cells. The experimental results revealed that a concentration of 50,000 ng/mL significantly inhibited the proliferation of renal tubular cells. Consequently, in this investigation the stimulation concentration was set at 50 µg/mL, ensuring that the chosen condition reliably reflects the inhibitory effects observed. This methodical approach not only underscores the precision of the experimental design but also enhances the overall validity and reproducibility of the study's findings. One-way ANOVA is used for comparisons of three or more groups. Data are presented as mean ± SD. Statistical significance is indicated as follows: *****p* < 0.0001.


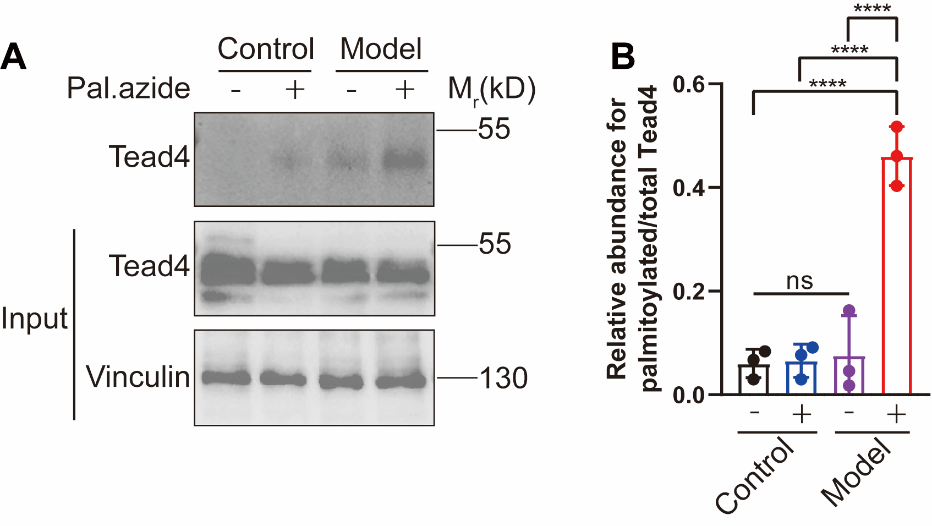


**Fig. S3.** Assessment of TEAD4 palmitoylation in the pmTECs of bovine serum albumin (BSA) nephritis mice. (A-B) To determine the palmitoylation status of TEAD4, pmTECs were isolated from control and model mice. Subsequent Click-iT analysis revealed that TEAD4 palmitoylation was significantly potentiated pmTECs from the model group relative to the control group (n=3). One-way ANOVA is used for comparisons of three or more groups. Data are presented as mean ± SD. Statistical significance is indicated as follows: ns, not significant; *****p* < 0.0001.


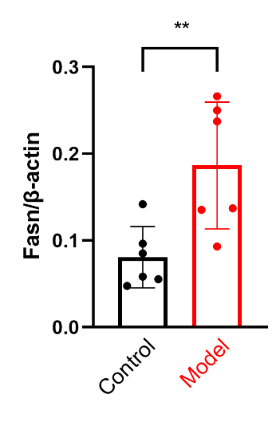

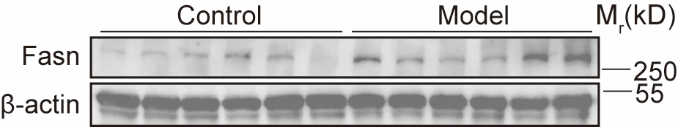
**A B**


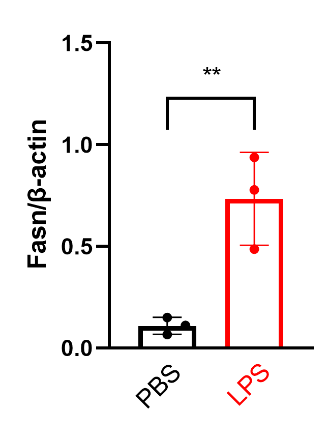
**C D**


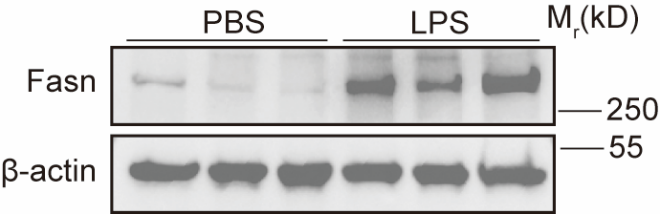


**Fig. S4.** Expression of FASN in BSA nephritis and LPS-induced pmTECs models. (A-B) In the BSA nephritis mice, FASN expression in renal tissues was markedly upregulated. This finding was validated using WB analysis, in which FASN protein levels were measured with β-actin serving as the loading control (n=6). (C-D) In an inflammatory cell model, FASN expression was significantly elevated in pmTECs following LPS stimulation (n=3). All statistical analyses were conducted using Student’s t test, and the data are presented as mean ±SD, with significance defined as: ***p* < 0.01.


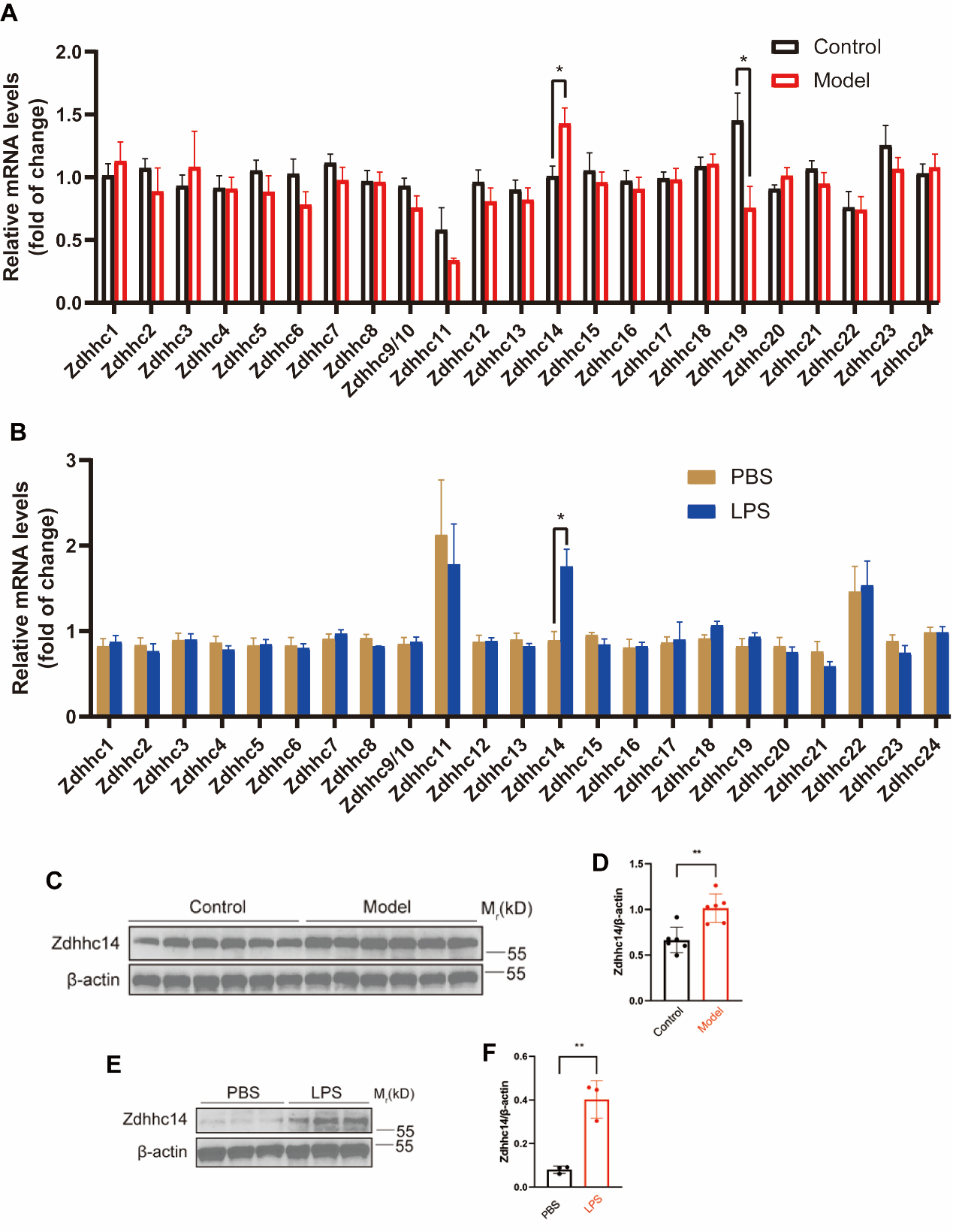


**Fig. S5.** Expression profile of the ZDHHC family in the BSA nephritis mice and in LPS-stimulated renal TECs. (A) qRT-PCR assays of the ZDHHC family in renal tissue from the BSA nephritis mice revealed that ZDHHC14 was significantly upregulated at the mRNA level compared to controls (n=6). (B) Consistent with the in vivo findings, qRT-PCR analysis in LPS-stimulated pmTECs revealed a parallel and significant elevation in ZDHHC14 mRNA expression relative to PBS-treated cells (n=3). (C-D) Quantitative and qualitative analyses of kidney specimens from the BSA nephritis mice revealed a marked increase in ZDHHC14 expression. This finding was validated using WB analysis, in which ZDHHC14 protein levels were measured with β-actin serving as the loading control (n=6). (E-F) In pmTECs stimulated with LPS, the expression of ZDHHC14 was significantly elevated (n=3). Comparisons between two groups are performed using Student’s t-test, whereas differences among three or more groups are assessed by one-way analysis of variance (ANOVA). All results are expressed as mean ± standard deviation. Statistical significance is denoted as follows: **p* < 0.05; ***p* < 0.01.

**A B**


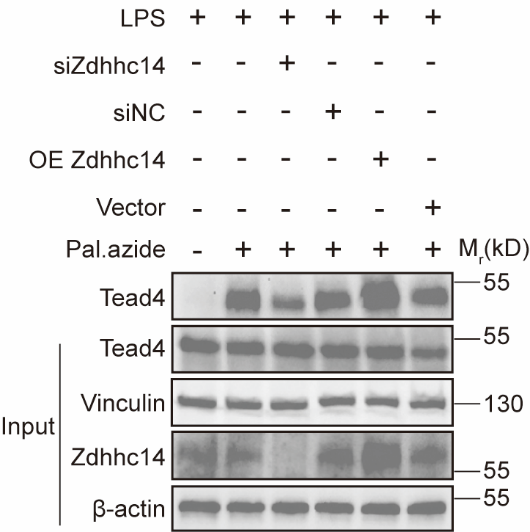

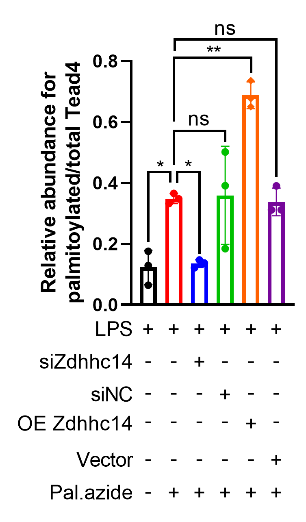


**Fig. S6.** The relationship between ZDHHC14 and TEAD4 palmitoylation. (A-B) Consistent with the ABE assay, the Click-iT assay demonstrated that ZDHHC14 knockdown (via siRNA) reduced, while ZDHHC14 overexpression increased, TEAD4 palmitoylation levels in inflamed renal TECs (n=3). All data are presented as mean ± SD and analyzed using one-way ANOVA. Statistical significance is indicated as follows: ns, not significant; **p* < 0.05; ***p* < 0.01.


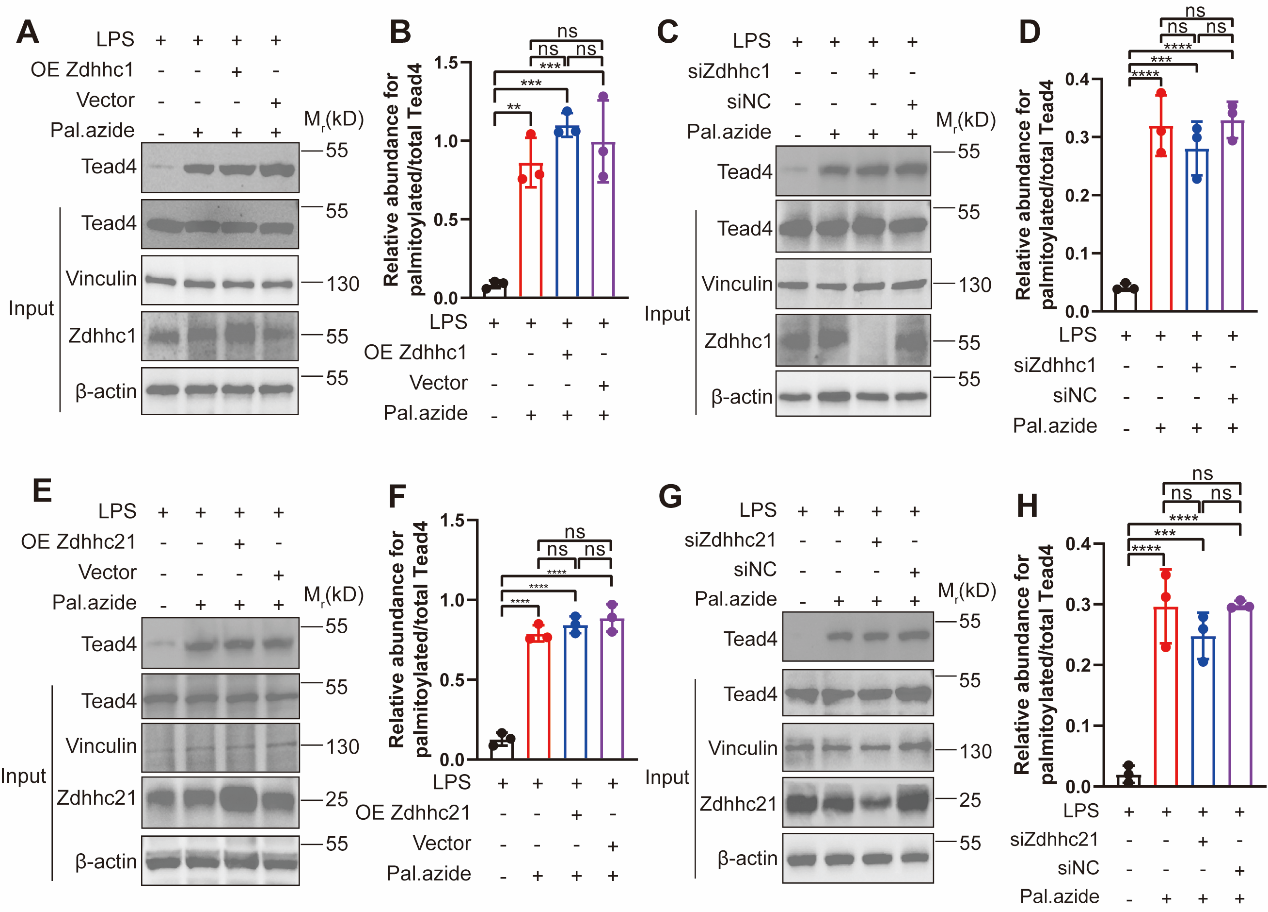


**Fig. S7.** Investigating the roles of ZDHHC1 and ZDHHC21 in regulating TEAD4 palmitoylation. (A-D) Prompted by a correlational analysis of renal transcriptome data from BSA nephritis mice, ZDHHC1 was examined as a potential regulator. However, neither siRNA-mediated knockdown (A-B) nor overexpression (C-D) of ZDHHC1 significantly altered the palmitoylation status of TEAD4 (n=3). (E-H) Similarly, ZDHHC21 was assessed as another putative candidate; yet, both its knockdown (E-F) and overexpression (G-H) had no discernible effect on TEAD4 palmitoylation levels (n=3). One-way ANOVA is used for comparisons of three or more groups. Data are presented as mean ± SD. Statistical significance is indicated as follows: ns, not significant; ***p* < 0.01; ****p* < 0.001; *****p* < 0.0001.


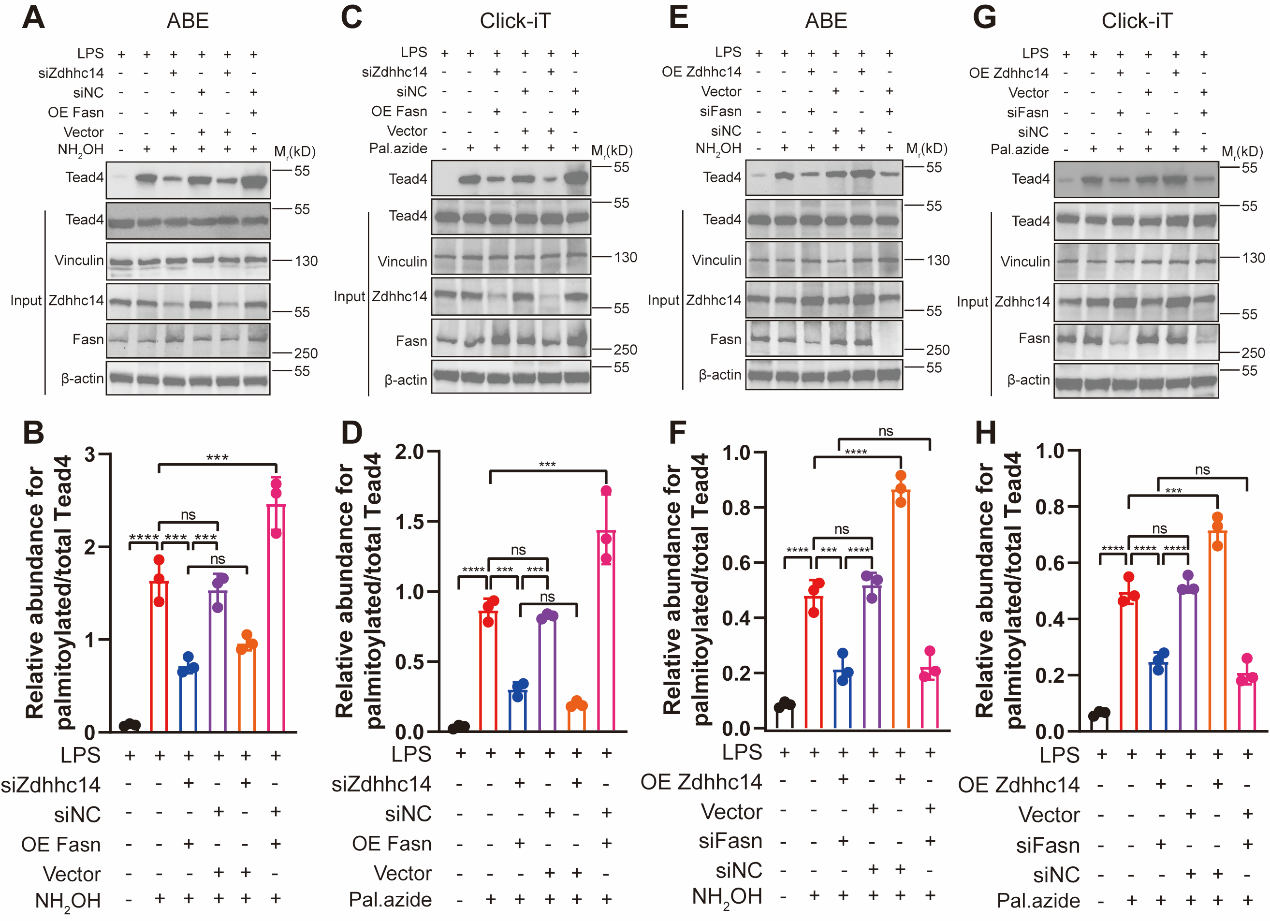


**Fig. S8.** FASN and ZDHHC14 are both indispensable for TEAD4 palmitoylation. (A-D) Data collectively derived from both ABE and Click-iT assays demonstrated that in cells with ZDHHC14 knockdown, concurrent overexpression of FASN was insufficient to restore TEAD4 palmitoylation, which remained at a significantly reduced level (n=3). (E-H) Conversely, in the context of FASN knockdown, overexpressing the writer enzyme ZDHHC14 likewise failed to rescue the suppression of TEAD4 palmitoylation (n=3). One-way ANOVA is used for comparisons of three or more groups. Data are presented as mean ± SD. Statistical significance is indicated as follows: ns, not significant; ****p* < 0.001; *****p* < 0.0001.


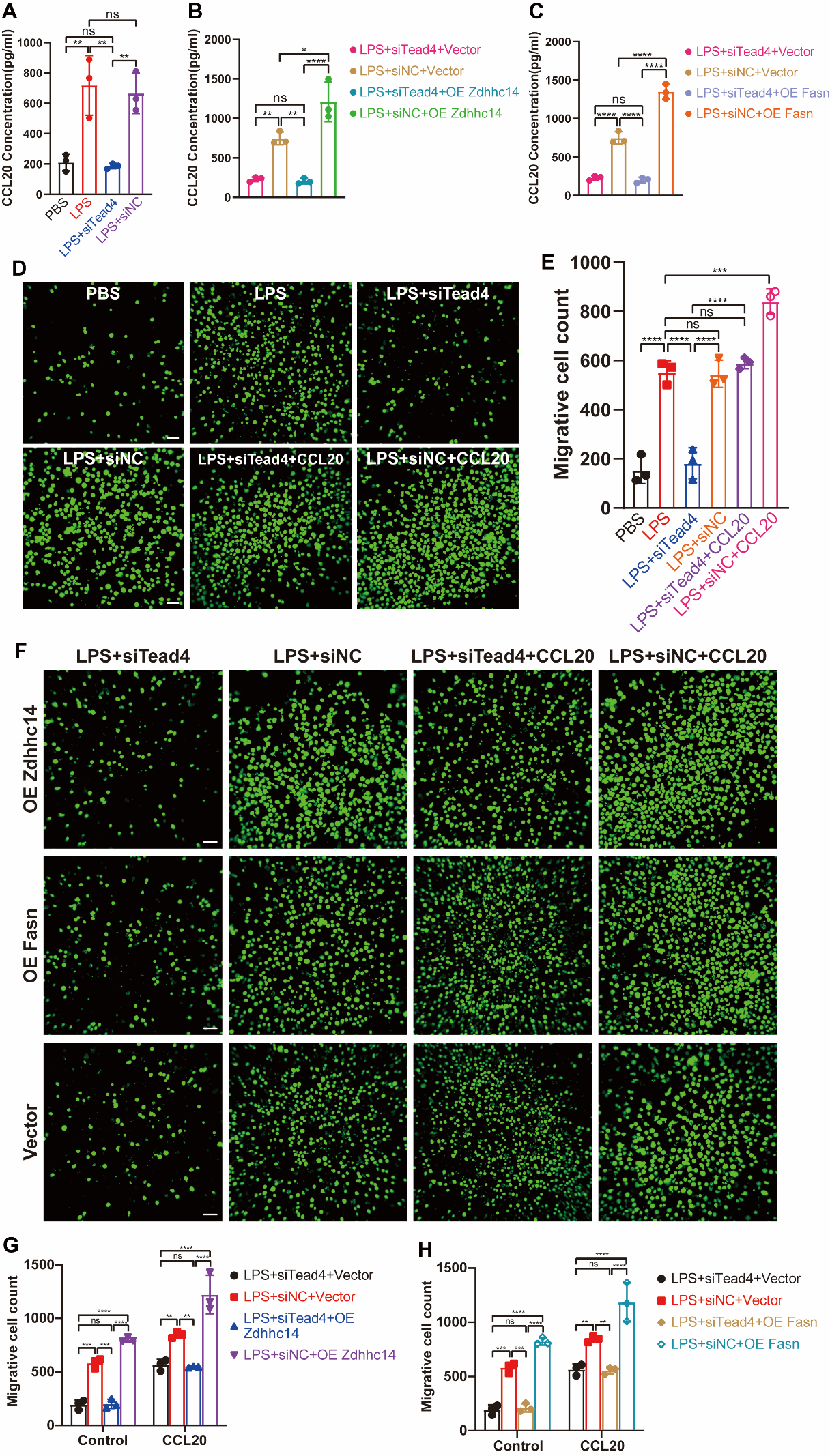


**Fig. S9.** The pro-inflammatory effects of FASN and ZDHHC14 are mediated through TEAD4. (A-C) In LPS-stimulated pmTECs, knockdown of TEAD4 abrogated CCL20 expression (A). Critically, this reduction in CCL20 was not reversed by the concurrent overexpression of either ZDHHC14 (B) or FASN (C), establishing that TEAD4 acted downstream to regulate CCL20 production. (D-H) This molecular change translated to a functional outcome: TEAD4 knockdown significantly impaired the chemotactic migration of Th17 cells toward inflamed renal TECs (D-E). Consistent with the gene expression data, this migratory defect could not be rescued by overexpressing ZDHHC14 or FASN (F-H). However, the migration was fully restored by the addition of exogenous CCL20 in all TEAD4-knockdown conditions (D-H), confirming CCL20 as the key downstream effector chemokine (n=3). One-way ANOVA is used for comparisons of three or more groups. Data are presented as mean ± SD. Statistical significance is indicated as follows: ns, not significant; **p* < 0.05; ***p* < 0.01; ****p* < 0.001; *****p* < 0.0001.

**References**

1. Wu B, Zhang S, Guo Z, Bi Y, Zhou M, Li P, et al. The TGF-β superfamily cytokine Activin-A is induced during autoimmune neuroinflammation and drives pathogenic Th17 cell differentiation. *Immunity*. 2021;54(2).

2. Alseekh S, Aharoni A, Brotman Y, Contrepois K, D'Auria J, Ewald J, et al. Mass spectrometry-based metabolomics: a guide for annotation, quantification and best reporting practices. *Nat Methods*. 2021;18(7):747-756.

3. Kim D, Langmead B, Salzberg SL. HISAT: a fast spliced aligner with low memory requirements. *Nat Methods*. 2015;12(4):357-360.

4. Mortazavi A, Williams BA, McCue K, Schaeffer L, Wold B. Mapping and quantifying mammalian transcriptomes by RNA-Seq. *Nat Methods*. 2008;5(7):621-628.

5. Pertea M, Kim D, Pertea GM, Leek JT, Salzberg SL. Transcript-level expression analysis of RNA-seq experiments with HISAT, StringTie and Ballgown. *Nat Protoc*. 2016;11(9):1650-1667.

6. Pertea M, Pertea GM, Antonescu CM, Chang T-C, Mendell JT, Salzberg SL. StringTie enables improved reconstruction of a transcriptome from RNA-seq reads. *Nat Biotechnol*. 2015;33(3):290-295.

7. Robinson MD, McCarthy DJ, Smyth GK. edgeR: a Bioconductor package for differential expression analysis of digital gene expression data. *Bioinformatics*. 2010;26(1):139-140.

8. Robinson MD, Oshlack A. A scaling normalization method for differential expression analysis of RNA-seq data. *Genome Biol*. 2010;11(3):R25.

9. Wu T, Hu E, Xu S, Chen M, Guo P, Dai Z, et al. clusterProfiler 4.0: A universal enrichment tool for interpreting omics data. *Innovation (Camb)*. 2021;2(3):100141.
